# Label-Free and High-Throughput Quantification of Nanoparticle–Cell Interactions at the Single-Cell Level with Flow Cytometry

**DOI:** 10.1021/acs.analchem.5c08235

**Published:** 2026-06-10

**Authors:** Mobina Mohammadnejad, Majood Haddad, Alex N. Frickenstein, Arianna Dambold, Vinit Sheth, Jezan Alexandre, Nathan Means, James Bowman, Kavita Belligund, Hunter Moss, Jeesoo Park, Yuxin He, Stefan Wilhelm

**Affiliations:** † Stephenson School of Biomedical Engineering, 6187 University of Oklahoma, Norman, Oklahoma 73019, United States; ‡ 6190 Oklahoma Medical Research Foundation, Oklahoma City, Oklahoma 73104, United States; § Institute for Biomedical Engineering, Science, and Technology (IBEST), University of Oklahoma, Norman, Oklahoma 73019, United States; ∥ Stephenson Cancer Center, University of Oklahoma, Oklahoma City, Oklahoma 73104, United States; ⊥ Harold Hamm Diabetes Center, University of Oklahoma, Oklahoma City, Oklahoma 73014, United States; # Materials Science and Engineering Program, University of Oklahoma, Oklahoma City, Oklahoma 73019, United States

## Abstract

Understanding nanoparticle–cell interactions at the single-cell level is essential for designing next-generation nanomedicines. Here, we explored flow cytometry as a single-cell technique for label-free quantification of nanoparticle–cell interactions. We demonstrated the use of conventional flow cytometry-based side-scattering signals to quantify interactions between nanoparticles and individual cells. We corroborated our findings qualitatively with optical super-resolution microscopy and quantitatively with elemental mass spectrometry. Using a broad, multiparameter workflow, we analyzed >10,000 single cells per minute to evaluate how nanoparticle size, composition, surface chemistry, and concentration affect cellular interactions, and we leveraged this approach to quantify nanoparticle uptake kinetics at the single-cell level. We further validated our findings using super-resolution expansion microscopy and single-particle inductively coupled plasma mass spectrometry, and extended the applicability of this workflow to mixed-cell and coculture in vitro cell models. Our demonstrated workflows enable a quantitative understanding of nanoparticle–cell interactions for the rational design of next-generation nanomedicines that are safer, more effective, and more efficient.

## Graphical abstract

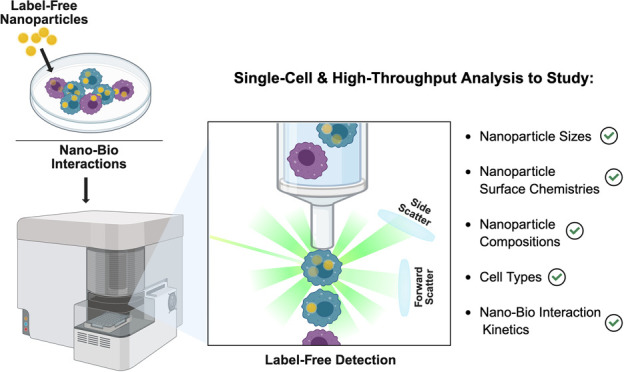

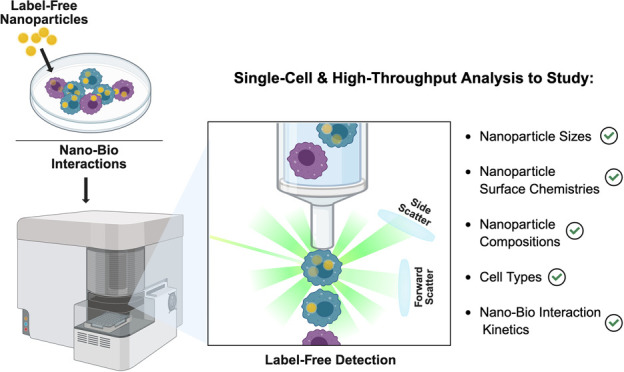

## Introduction

Engineering safer, more effective nanomedicines requires a better quantitative understanding of nanoparticle–cell interactions at the single-nanoparticle and single-cell levels.^1,2^ Despite extensive research efforts, studies have shown that only a limited amount of the administered nanoparticle dose reaches solid tumors.^3^ The therapeutic efficacy of nanoparticle-based systems, therefore, depends on the successful delivery and internalization of nanoparticles by target cells.^4^ This limited delivery efficiency highlights the need for systematic approaches to better understand how nanoparticles interact with cells and how physicochemical nanoparticle design parameters influence cellular internalization.

This challenge is exacerbated by cellular heterogeneity. Even cells within the same population can exhibit significant differences in nanoparticle uptake and kinetics.^5–7^ Capturing this variability requires analytical methods capable of quantifying nanoparticle–cell interactions at the single-cell level. An ideal method should enable label-free nanoparticle detection, preserve nanoparticle surface chemistry, and provide high-throughput single-cell analysis.^8–12^

Current methods for evaluating nanoparticle–cell interactions are often labor-intensive, require specialized equipment, or have limited throughput. For example, optical microscopy techniques typically require fluorescence and other labeling strategies, which may alter nanoparticle surface chemistry and, in turn, their interactions with cells. Microscopy is further limited in throughput and can be challenging to apply at scale.^1,13^ Quantitative single-nanoparticle and single-cell analyses by inductively coupled plasma mass spectrometry (ICP-MS) are also typically labor-intensive, destructive, and have limited suitability for real-time analysis.^5,13–18^

Flow cytometry offers a potential solution to many of these limitations. Flow cytometers are widely available in biomedical research environments. These instruments can analyze thousands of single cells per minute, making them an ideal tool for high-throughput evaluation of complex cell populations.^1,19^ In addition to fluorescence, flow cytometers measure how cells scatter incident light, producing optical signals such as forward scatter (FSC) and side scatter (SSC). While FSC is commonly associated with cell size, SSC correlates with cellular complexity and granularity and is particularly sensitive to intracellular structural changes. As cells interact with nanoparticles, including gold nanoparticles (AuNPs) and other inorganic nanomaterials, measurable changes in SSC signals can occur. Prior SSC-based studies have demonstrated that these changes can be used to qualitatively detect nanoparticle uptake and, more recently, to quantitatively estimate the number of cell-associated gold and silver nanoparticles when SSC measurements are combined with ICP-MS validation in single cell-type systems.^20–24^

In this study, we systematically evaluated SSC-based flow cytometry for the high-throughput, label-free detection of nanoparticle–cell interactions across different nanoparticle types, sizes, concentrations, and cell lines, with nanoparticle–cell interactions primarily referring to nanoparticle internalization. Building on prior studies that established SSC as a tool for detecting nanoparticle presence and, more recently, as a quantitative readout of cellular nanoparticle content when combined with ICP-MS calibration for gold and silver nanoparticles in single-cell type systems,^24–26^ we extended this approach into a broader multiparameter experimental workflow. Unlike these prior studies, which were primarily focused on either qualitative detection of nanoparticle presence, genotoxicity screening, or quantitative SSC-ICP-MS calibration for specific nanoparticle–cell line combinations,^20,22–27^ the present work systematically evaluated how specific nanoparticle design parameters, such as size, surface chemistry, and composition, influence cellular uptake across multiple nanoparticle types and concentrations. We further examined nanoparticle internalization kinetics and validated our findings with optical super-resolution microscopy and elemental mass spectrometry. Extending our analysis to mixed-cell and coculture models, we demonstrated that this workflow can detect context-dependent differences in nanoparticle uptake in more physiologically relevant *in vitro* cellular models. Together, this proof-of-concept study demonstrates the application of high-throughput, label-free flow cytometry as a practical screening tool for nanomedicine and nanoparticle-based delivery strategies.

## Experimental Section

### Gold Nanoparticle Synthesis and Surface Modification

AuNPs with diameters of 14, 40, 65, and 100 nm were synthesized using citrate-based seed-mediated growth methods.^15,28,29^ We prepared the 14 nm AuNP seeds via a modified Turkevich method, in which chloroauric acid was reduced by sodium citrate under boiling conditions.^29^ We achieved the synthesis of larger AuNPs using a seed-mediated approach adapted from Perrault et al., guided by predictive growth parameters, with hydroquinone serving as the reducing agent.^15,28^ We purified the AuNPs by centrifugation to remove excess reactants and secondary nucleation products. All synthesized AuNPs were stored at 4 °C before further use. We performed nanoparticle surface modification to improve colloidal stability using 10-kDa poly­(ethylene glycol) (PEG) at a target surface density of 7 ligands nm^–2^.^16^ We measured the AuNPs’ hydrodynamic diameter and molar concentration before coating to determine the required ligand amounts. Following incubation, we purified the PEGylated nanoparticles by centrifugation and resuspended them in a sodium citrate buffer containing Tween20 as a surfactant. We prepared 30 nm AgNPs that were PEGylated using a similar procedure with 5-kDa PEG. Heparosan (HEP)-coated AuNPs were prepared by modifying citrate-stabilized 100 nm AuNPs with OPSS-modified heparosan polysaccharides at the same target ligand density based on our previously established procedure.^30^ We performed the coating under acidic conditions with stepwise salt addition to promote surface grafting. Coated AuNPs were purified by repeated centrifugation and resuspension and stored at 4 °C until use. More detailed step-by-step procedures are available in the Supporting Information.

### Nanoparticle Characterization

We measured the AuNPs hydrodynamic diameter and polydispersity index (PDI) with dynamic light scattering (DLS). Ultraviolet–visible (UV–vis) spectrophotometry was used to record extinction spectra and calculate molar concentrations using the Beer–Lambert law and literature-reported molar extinction coefficients. (Table S1)^28,31^ Transmission electron microscopy (TEM) was used to assess core diameter distributions, which were quantified using ImageJ, assuming spherical geometry.

We performed single-particle inductively coupled plasma mass spectrometry (SP-ICP-MS) to determine nanoparticle mass and size distributions at the single-particle level. Diameters were calculated from measured particle mass assuming bulk density and spherical geometry (Table S2).^15–18^

### Cell Culture

We cultured RAW 264.7 murine macrophages in DMEM, while DC 2.4 dendritic cells and 4T1 murine breast cancer cells were cultured in RPMI-1640, each supplemented with fetal bovine serum (FBS) and penicillin/streptomycin (P/S). Cells were incubated at 37 °C in a humidified 5% CO_2_ atmosphere and passaged at 80–90% confluency. Cells were collected by centrifugation under cell-type-specific conditions before experimentation.

### Cell Treatments with Nanoparticles

Cells were seeded in 12-well plates and allowed to adhere for 22–24 h before treatment. Nanoparticle concentrations were determined by UV–vis spectrophotometry. We treated the Cells with nanoparticles for up to 24 h to allow for uptake and internalization. Experiments were conducted to assess concentration-, size-, surface chemistry-, cell type-, and time-dependent nanoparticle uptake in monoculture, mixed-cell, and coculture models. Final nanoparticle concentrations and exposure times are specified for each experiment in the Supporting Information.

### Fixation and Staining

For confocal microscopy, cells were fixed with paraformaldehyde, quenched with sodium borohydride and glycine, and stained with nuclear and membrane markers (DAPI and WGA). For flow cytometry, cells were fixed and washed before analysis. Cell viability was determined using Ghost Dye Violet 510, a fixable amine-reactive viability dye. For mixed-cell and coculture experiments, membrane dyes of DiI and DiD were used to distinguish individual cell populations. Expansion microscopy samples were pan-stained using an NHS-ester BP 488 dye following gel expansion.

### CLSM and Expansion Microscopy

Cells were imaged using a CLSM (Zeiss LSM780). Z-stack images were collected and processed using Zen 2010 and ImageJ software. Expansion microscopy was performed using a Magnify-based protocol, involving gel embedding, homogenization, fluorescent labeling, and expansion with water.^32^

### Flow Cytometry

Flow cytometry was performed using a spectral flow cytometer equipped with violet, blue, and red lasers. Instrument gains were optimized for each cell type and held constant across experimental conditions to enable direct comparison of scattering profiles. Data were acquired using SpectroFlo and analyzed using FlowJo software.

### Statistical Analysis and Figure Preparation

Statistical analyses and data visualization were performed using GraphPad Prism. Final figures were assembled using GraphPad Prism and Adobe Illustrator.

## Results and Discussion

### Nanoparticle Physicochemical Characterization

We selected 100 nm gold nanoparticles (AuNPs) as our primary model system because they offer a well-balanced combination of physicochemical and biological properties relevant to nanobio interactions.^33^ Nanoparticles in this size range exhibit efficient cellular uptake and mimic the sizes of clinically used nanoparticles, such as liposomes.^34–37^ Gold nanoparticles also exhibit chemical stability, resistance to degradation, cost-effectiveness, and ease of synthesis, enabling high-yield fabrication with consistent physicochemical properties.^15,16^ The unique optical properties, particularly the strong light absorption and scattering, enable the label-free detection of AuNPs.^38–40^ Additionally, the AuNPs can be quantified using elemental analysis techniques, such as ICP-MS, providing complementary data that validate optical measurements. Finally, the biocompatibility and minimal cytotoxicity make AuNPs an ideal model system for studying nanoparticle–cell interactions.^41–44^

To optimize the performance of these nanoparticles for biological applications, PEGylation was used to enhance colloidal stability and prevent nanoparticle aggregation upon cellular exposure. In addition to improving stability, PEG also reduces nonspecific protein adsorption in biological media. However, although PEG exhibits antiadsorptive properties, it does not fully inhibit cellular interactions and internalization. Nanoparticles can still be internalized through established endocytic pathways.^45–48^ For the 100 nm AuNPs, we selected 10-kDa PEG because its relatively long polymer chains create a dense steric layer on the nanoparticles’ surface to enhance colloidal stability.^17,49–52^

To ensure the synthesized AuNPs reflect the targeted size and quality, we characterized them using UV–vis spectrophotometry, TEM, DLS, electrophoretic mobility (Zeta potential), and SP-ICP-MS. These complementary techniques provide a comprehensive assessment of the nanoparticles’ physicochemical properties, including size distribution, composition, surface charge, and colloidal stability. The UV–vis spectrum of 100 nm AuNPs (Figure S1) highlights the characteristic surface plasmon resonance absorption peak of ∼575 nm. TEM images confirmed the nanoparticle diameter and size distribution (Figure 1A, B). We assumed a spherical nanoparticle geometry, a convention widely used in the literature and adopted in this study.^16,17,28^ The size analysis of 612 nanoparticles yielded a mean diameter of 101.6 ± 8.9 nm, which closely aligns with the target size of 100 nm, indicating a narrow size distribution (Figure 1B). DLS measurements showed an increase in hydrodynamic diameter (HDD) from 124.8 ± 0.4 nm for citrate-coated AuNPs to 143.4 ± 1.7 nm after PEGylation (Figure 1C), with a PDI < 0.1, corroborating narrow size distributions and colloidal stability. Zeta potential analysis further supported these findings, showing a shift from −26.8 ± 0.9 mV for citrate-coated AuNPs to 1.5 ± 0.9 mV for PEGylated AuNPs (Table S3).^53^ We further used SP-ICP-MS to corroborate the mean nanoparticle diameters for citrate-coated (101.1 ± 7.3 nm) and PEG-coated (101.3 ± 7.2 nm) AuNPs (Figure 1D), indicating that PEGylation did not significantly alter the core nanoparticle size. These results confirm that the synthesized nanoparticles exhibit narrow size distributions and strong colloidal stability. The low polydispersity values and consistent size measurements across multiple characterization techniques indicate that the nanoparticles remain well-dispersed under the experimental conditions, minimizing potential aggregation that could otherwise lead to uneven cellular exposure.

**Figure 1:**
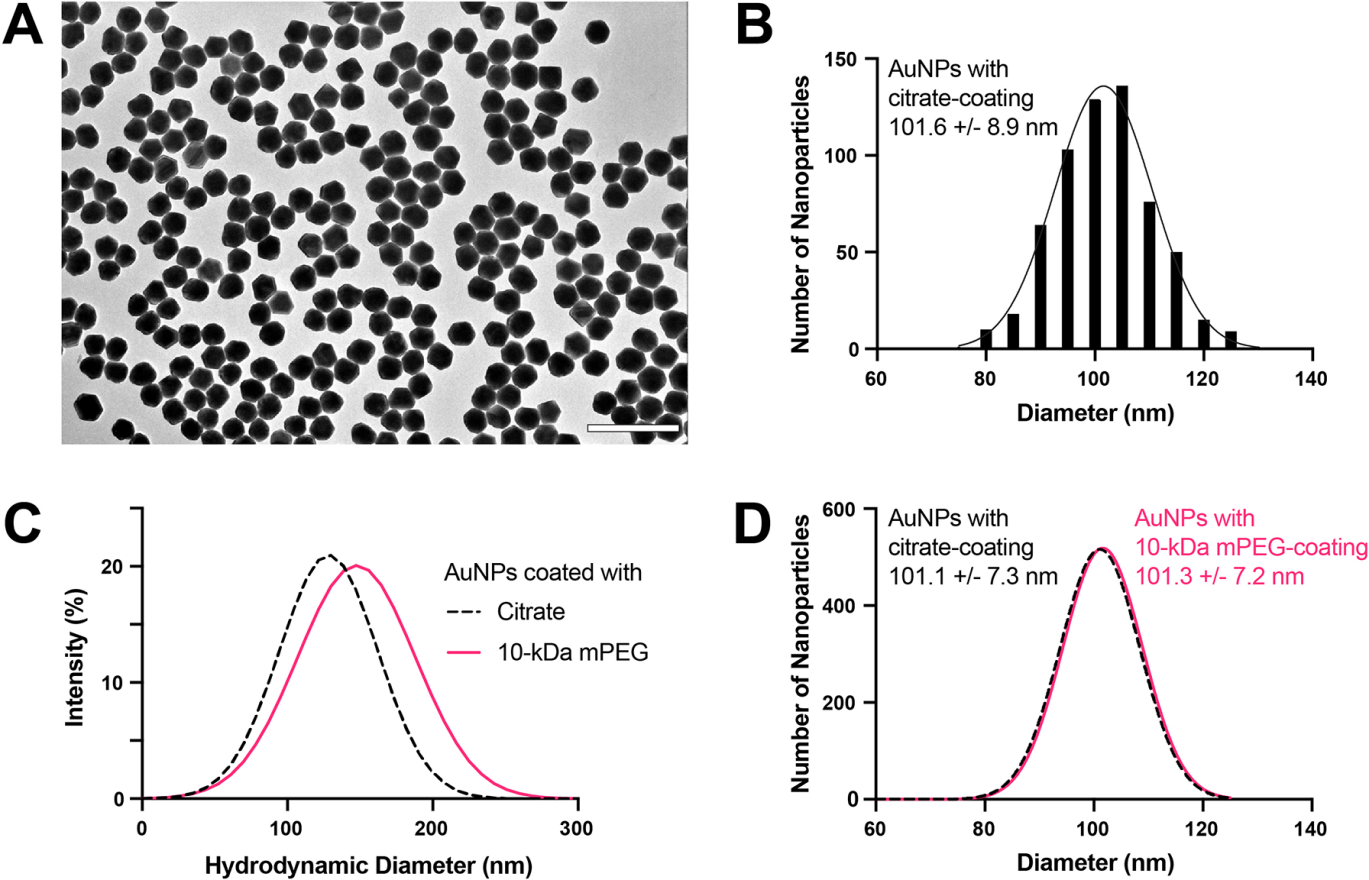
Physicochemical characterization of 100 nm gold nanoparticles (AuNPs). (A) Representative TEM image of the synthesized 100 nm AuNPs. The scale bar indicates 400 nm. (B) Nanoparticle size distribution analysis based on TEM images. The size distribution histogram is fitted with a Gaussian curve (black line). The mean diameter is 101.6 ± 8.9 nm (standard deviation, *n* = 612). (C) DLS characterization of hydrodynamic diameter (HDD) for citrate-coated 100 nm AuNPs (black, average HDD is 124.8 nm; PDI is 0.065) and PEG-coated 100 nm AuNPs (red, average HDD is 143.3 nm, PDI = 0.045). (D) Results of SP-ICP-MS to compare the core size distributions of citrate-coated (black, 101.1 ± 7.3 nm, *n* = 1,900) and PEGylated (pink, 101.3 ± 7.2 nm, *n* = 1,900) AuNPs.

### Establishing Label-Free Detection of Nanoparticle-Cell Interactions at the Single-Cell Level

We performed a series of experiments to establish our label-free approach for quantifying nanoparticle–cell interactions. We incubated RAW 264.7 murine macrophages with 100 nm AuNPs and confirmed the nanoparticle intracellular localization using confocal laser scanning microscopy (CLSM). We selected RAW 264.7 cells because they are phagocytic and widely used in preclinical studies. Cells treated with AuNPs exhibited light-scattering signals, which were absent in untreated controls, confirming AuNP internalization by the cells (Figure 2A).^54,55^

**Figure 2:**
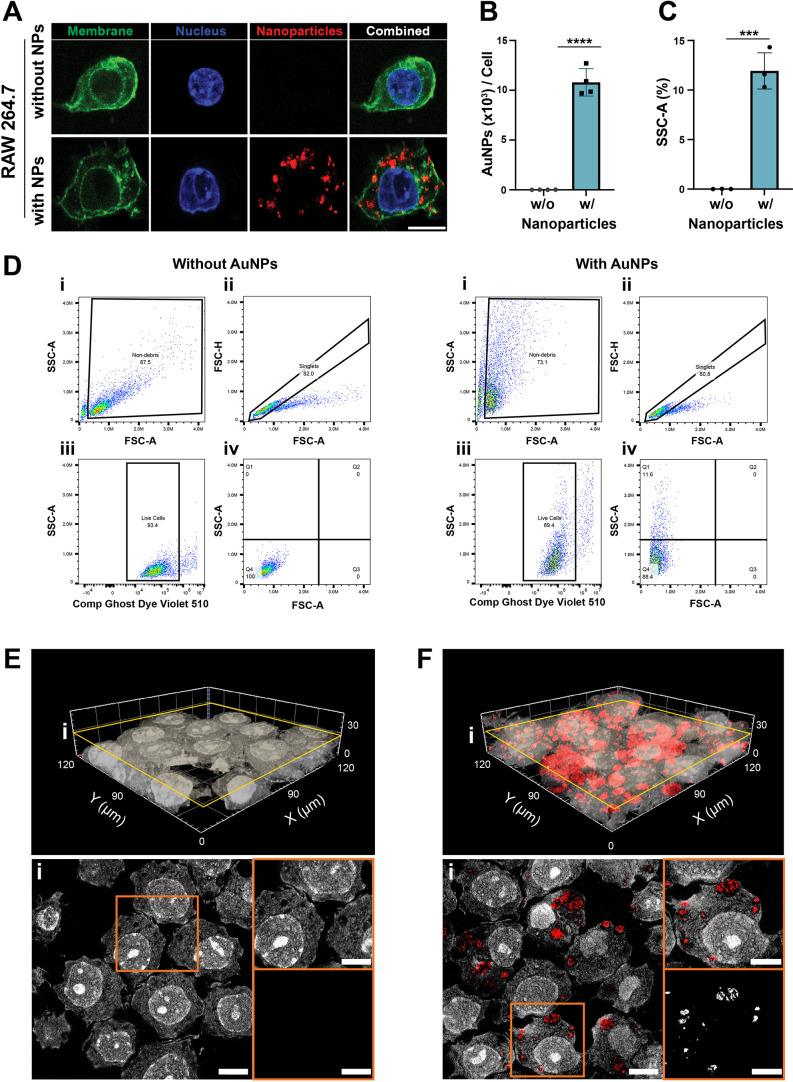
Label-free detection of 100 nm gold nanoparticle (AuNP) uptake in RAW 264.7 macrophages. (A) Representative CLSM images of RAW264.7 murine macrophages without and with AuNPs. The cell membranes were labeled with fluorescent wheat germ agglutinin (WGA-CF488A, green). The cell nuclei DNA was stained with 4′,6-diamidino-2-phenylindole (DAPI, blue). The red color corresponds to light scattering signals from 100 nm AuNPs. The scale bar represents 10 μm. (B) ICP-MS in batch mode was used to quantify the average cellular interactions of 100 nm AuNPs with RAW 264.7; unpaired *t* test (****p < 0.0001; unpaired *t* test; *n* = 4, mean ± standard deviation). (C) Flow cytometry was used to quantify SSC-A (%); (***p < 0.0005; unpaired *t* test; *n* = 3, mean ± standard deviation). (D) Flow cytometry gating strategy (i) Debris was excluded using FSC-A vs SSC-A gating. (ii) Singlets were selected using FSC-H vs FSC-A. (iii) Live cells were gated using Ghost Dye Violet 510-A to exclude dead cells. (iv) Q1 was chosen as the area of interest to show the increase in SSC-A following the AuNPs treatment. (E, F) Expansion microscopy (ExM) images of cells with CLSM-based 3D reconstructions and fluorescent NHS-ester bulk (pan) staining (BP Fluor 488-NHS, gray). The yellow-outlined x/y slices correspond to the areas shown in micrographs E, i and F, i. Digital magnifications of the regions of interest are shown (orange outline), first with an overlay of both channels (NHS-ester pan stain (gray) and light scattering (red)). Next, the nanoparticle light scattering signal is shown in grayscale. The scale bars represent 20 μm.

To corroborate these findings, we used ICP-MS to quantify the number of nanoparticles per cell, revealing ∼ 10,000 AuNPs per cell. This result was significantly higher than that of the controls (*p* < 0.0001, Figure 2B). The measured uptake of approximately 10,000 AuNPs per cell falls within ranges reported in previous nanoparticle–cell interaction studies.^56–58^ Macrophages exhibit high capacities for nanoparticle internalization due to their phagocytic function, with uptake levels commonly ranging from thousands to tens of thousands of nanoparticles per cell, depending on nanoparticle properties and exposure conditions.^57,58^

We then evaluated flow cytometry as a high-throughput, label-free method for detecting nanoparticle–cell interactions at the single-cell level. We analyzed the side-scattering signal area (SSC-A), a parameter that reflects cellular granularity and internal structural complexity. This value represents the total side-scattered light signal collected as each cell passes through the laser interrogation point, measuring the cumulative light scattering generated by intracellular structures.^20,27,59,60^

As the AuNPs exhibit strong optical scattering properties due to their localized surface plasmon resonance, their interaction and accumulation within cells can increase the total side-scattered light detected.^61^ Consequently, nanoparticle-associated scattering can be detected as shifts in SSC-A distributions between untreated and nanoparticle-treated cell populations. To quantify these changes, we implemented the gating strategy to exclude debris, doublets, and dead cells (Figure 2D). We then defined a specific SSC region (Q1) as nanoparticle-positive cell events based on shifts observed in treated versus untreated cell samples. In this study, SSC-A (%) refers to the percentage of viable single cells exhibiting increased side-scatter signals relative to untreated controls (eq 1) and is defined as

1 SSC‐A(%)=(number of SSC‐positive
cells in region Q1/total viable
single cells)×100

While SSC-A(%) provides a convenient metric for detecting nanoparticle-associated changes in scattering, its sensitivity depends on the optical scattering properties of the nanoparticles and may decrease for very small nanoparticles or materials with inherently weak scattering signals, such as polymeric or lipid-based nanoparticles.

Using this approach, we observed that the percentage of nanoparticle-positive cells in region Q1 increased significantly in nanoparticle-treated samples compared to controls (*p* < 0.0005, Figure 2C). This shift from the low-SSC region (Q4) to the high-SSC region (Q1) indicates increased cellular complexity associated with nanoparticle uptake.

All experiments followed a consistent hierarchical gating workflow. This included debris exclusion (FSC-A vs SSC-A) (Figure 2D, (i), doublet exclusion (FSC-H vs FSC-A) (Figure 2D, (ii), and viability gating (Ghost Dye vs SSC-A) (Figure 2D, (iii), followed by final SSC-based analysis (FSC-A vs SSC-A). The final SSC gate (Figure 2D, (iv) was defined relative to the untreated control for each specific experimental model. Because different cell types exhibit distinct baseline morphologies and intrinsic SSC distributions, minor positional adjustments of this gate were required to accurately define the SSC-positive population. Once established using the corresponding control, the gate was held constant across all treatment groups in that experiment to ensure an unbiased comparison across experimental conditions.

Next, we used our unique expansion microscopy-based optical super-resolution 3D imaging workflow to further corroborate these results. We recently demonstrated that label-free 3D imaging of AuNPs within cells can be achieved using expansion microscopy.^54^ As a control, we used RAW 264.7 cells not exposed to AuNPs. As shown in Figure 2E, no light scattering signals of the expanded cells were observed upon 3D CLSM imaging. In contrast to this result, cells treated with AuNPs exhibited strong light-scattering signals, confirming the localization of nanoparticles within intracellular vesicular compartments (Figures 2F, S2). These unique super-resolution-based volumetric reconstructions demonstrate that the 100 nm AuNPs were not only internalized but also trafficked into intracellular vesicles, consistent with nanoparticle uptake pathways such as endocytosis and phagocytosis, as described in the literature.^54^

Although changes in SSC-A can also arise from alterations in cell morphology or activation states unrelated to nanoparticle uptake, multiple measurements in this study confirmed that the observed SSC shifts were associated with nanoparticle internalization. For example, CLSM imaging directly visualized intracellular AuNP-associated scattering signals, and ICP-MS provided quantitative evidence of substantial nanoparticle uptake per cell. In addition, cell viability assays showed no significant cytotoxicity under the experimental conditions, suggesting that the SSC-A increases were driven not by cellular stress or activation but by the accumulation of intracellular nanoparticles. (Figure S3) Together, CLSM, ICP-MS, and expansion microscopy 3D super-resolution imaging consistently validated the interactions between AuNPs and cells, establishing flow cytometry SSC-A as a reliable, label-free modality for quantifying nanoparticle–cell interactions at the single-cell level.

### Label-Free Quantification of Concentration-Dependent Nanoparticle-Cell Interactions

Next, we applied label-free flow cytometry to study how various nanoparticle concentrations affect nanoparticle–cell interactions. We incubated RAW 264.7 cells with varying molar concentrations of 100 nm AuNPs and measured the corresponding SSC-A(%) values. As shown in Figure 3A, the SSC-A(%) values increased in a nanoparticle concentration-dependent manner, exhibiting nonlinear behavior and approaching a plateau between 0.2 nM and 1 nM. Notably, even low nanoparticle molar concentrations (<0.1 nM) showed significant increases in SSC-A(%), indicating the sensitivity of the flow cytometry approach. We used a four-parameter logistic (4PL) regression model to fit the concentration–response relationship between SSC-A(%) and nanoparticle molar concentration. This model is widely used for modeling nonlinear, saturating dose–response data in biological systems.^62^ This model demonstrated a strong correlation between SSC-A (%) and nanoparticle molar concentration (R^2^ = 0.9004; eq 2). The model further suggests that RAW 264.7 cells exhibit limited nanoparticle-interaction capacity, beyond which increasing the nanoparticle concentration does not further increase the SSC-A value.

2 $$\mathrm{SSC‐A}\left(\%\right)=0.3057+\frac{21.31-0.3057}{1+{\left(\frac{\left[\mathrm{Nanoparticle}\right]}{0.08667}\right)}^{-1.983}}$$

**Figure 3:**
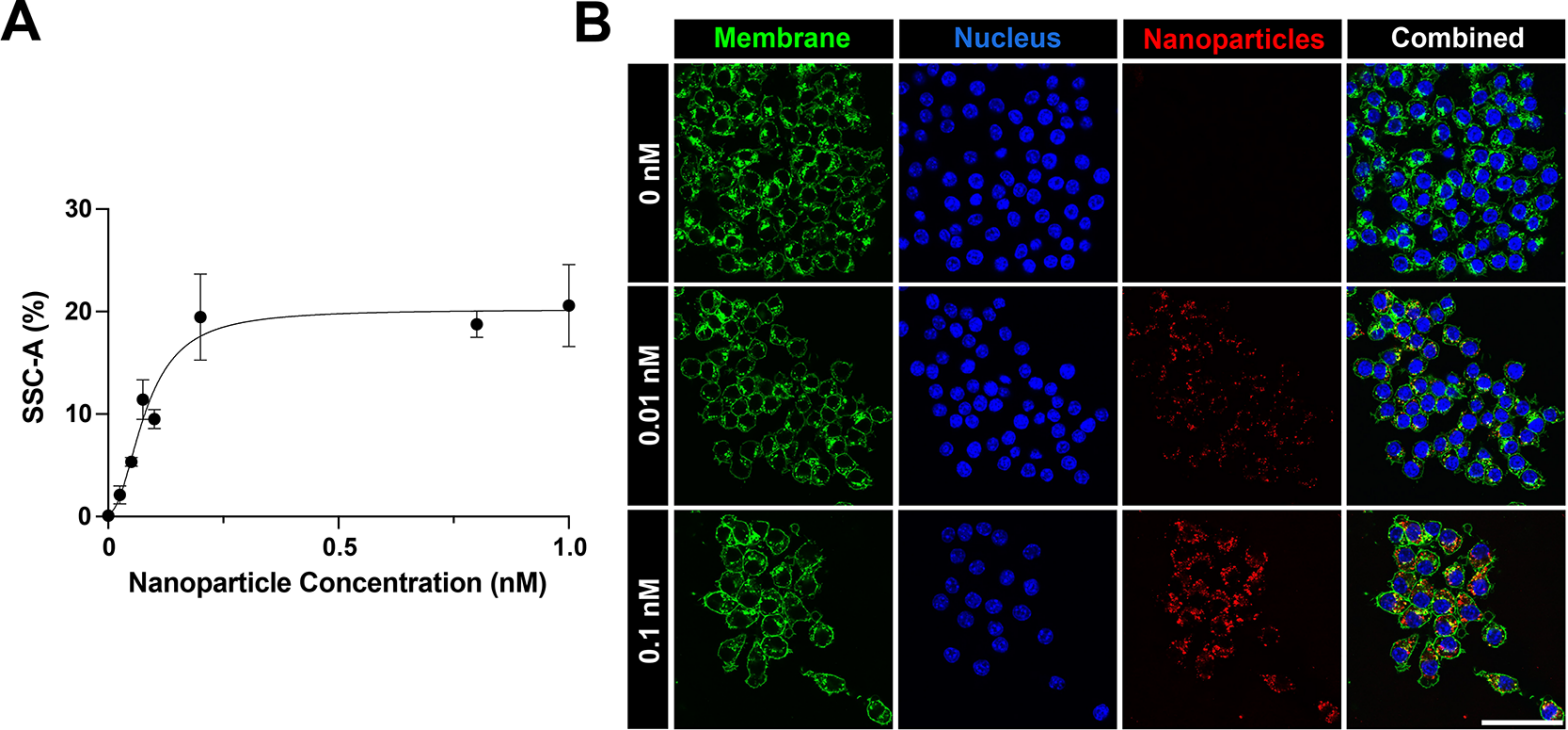
Quantification of the concentration-dependent nanoparticle interactions for RAW 264.7 Macrophages. (A) Concentration-dependent increase in SSC-A (%) after treatment with 100 nm AuNPs. RAW 264.7 murine macrophage cells were incubated with nanoparticles at concentrations up to 1.0 nM. The SSC-A(%) values were measured via flow cytometry, revealing an apparent concentration-dependent increase in nanoparticle–cell interactions. The solid line represents the 4PL regression model, which describes the saturating, nonlinear concentration–response relationship between SSC-A (%) and the nanoparticle molar concentration. Data points represent mean ± SD, *n* = 3. (B) Representative CLSM images of RAW 264.7 cells treated with 100 nm AuNPs at 0.01 nM and 0.1 nM and compared to control (0 nM), confirming a qualitative nanoparticle concentration-dependent increase in scattering signals (WGA-CF488A, green; DAPI, blue; nanoparticle light scattering, red). The scale bar represents 50 μm.

To verify the flow cytometry-based results, we used CLSM imaging to study the nanoparticle concentration-dependent light scattering behavior. We treated RAW 264.7 macrophage cells with two nanoparticle concentrations, i.e., 0.01 nM and 0.1 nM of AuNPs. As shown in Figure 3B, we observed significant nanoparticle light-scattering signals at both concentrations compared with the control group. Additionally, the detected light-scattering signals increased with nanoparticle molar concentration. The CLSM images were further quantified using ImageJ, and the normalized CLSM intensity was compared with normalized SSC-A measurements obtained from flow cytometry across the same concentration range (Figure S4). Both measurements exhibited strong linear concentration-dependent trends (R^2^ = 0.9926 for SSC-A and R^2^ = 0.9993 for CLSM intensity), supporting the use of SSC-A as a label-free indicator for quantifying concentration-dependent nanoparticle–cell interactions at the single-cell level.

### Label-Free Quantification of Nanoparticle-Cell Interactions across Various Nanoparticle Sizes, Compositions, Surface Chemistries, and Cell Types

Next, we tested the generalizability of our label-free flow cytometry approach for quantifying nanoparticle–cell interactions across nanoparticle sizes and compositions. We synthesized and characterized 14, 40, and 65 nm AuNPs (Figures S5–S7). Then we incubated RAW 264.7 macrophage cells with these nanoparticles. Flow cytometry analysis showed a significant increase in signal for both 40 and 65 nm AuNPs (Figure 4B, *p* < 0.0001), demonstrating sensitivity and reliable detection within this size range. However, this technique could not detect 14 nm AuNPs (Figure 4B). The reduced sensitivity observed for 14 nm AuNPs using flow cytometry can be attributed to their inherently weaker light-scattering properties due to their small size.

**Figure 4:**
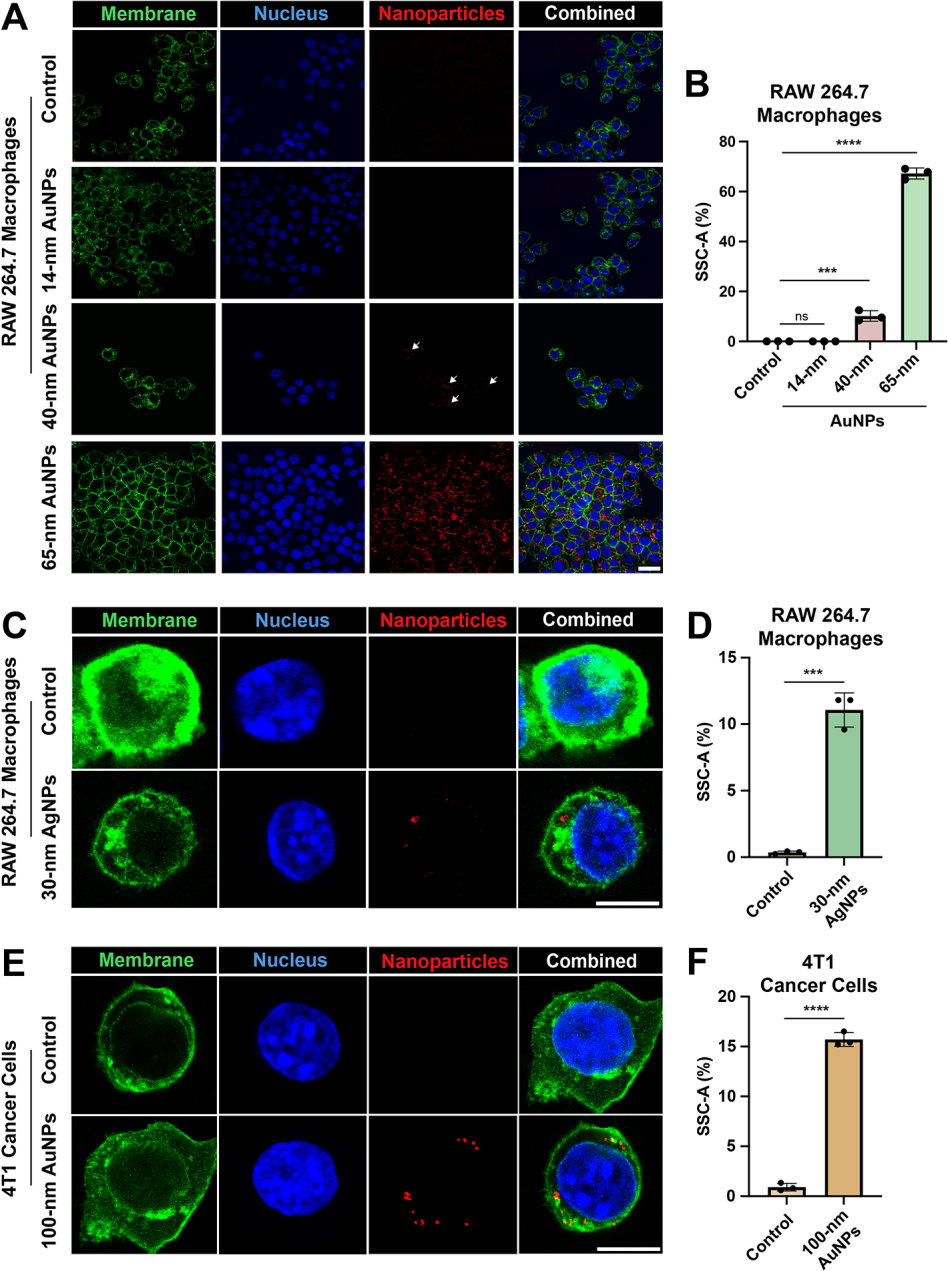
Label-free quantification of nanoparticle–cell interactions across various nanoparticle sizes, compositions, and cell types. (A) Representative CLSM images of RAW 264.7 macrophages treated with 14, 40, or 65 nm AuNPs, respectively, at a concentration of 1 nM compared to untreated control. Nuclei were stained with DAPI (blue), membranes with WGA-CF488A (green), and nanoparticles appear as a red signal. The white arrowheads indicate the locations of the nanoparticle signal. (B) Flow cytometry-based quantification of SSC-A(%) in RAW 264.7 macrophages treated with 14, 40, or 65 nm AuNPs (****p < 0.0001, ***p < 0.001, ns; one-way ANOVA, *n* = 3, mean ± standard deviation) at a concentration of 1 nM. (C) CLSM images of RAW 264.7 macrophages treated with 30 nm AgNPs, showing nanoparticle accumulation compared to control. (D) Flow cytometry-based quantification of SSC-A(%) in RAW 264.7 macrophages treated with 30 nm AgNPs confirmed a significant increase compared to control (*** *p <*0.001; unpaired *t* test, *n* = 3, mean ± standard deviation). (E) CLSM images of 4T1 murine breast cancer cells treated with 100 nm AuNPs compared to the control, showing AuNP uptake. (F) Flow cytometry quantification of SSC-A(%) in 4T1 cells treated with 100 nm AuNPs revealed a significant increase compared to control (****p < 0.0001; unpaired *t* test, *n* = 3, mean ± standard deviation). The scale bars represent 20 μm.

According to Mie theory, in the Rayleigh limit where particle diameter is much smaller than the incident wavelength, the scattering cross-section scales with the sixth power of particle diameter (σ_s_C_a_ ∝ d^6^), and therefore the measured scattering intensity is expected to follow the same size dependence (I ∝ d^6^) under constant illumination.^63^ As a result, decreases in particle size lead to orders-of-magnitude reductions in scattered intensity. For 14 nm AuNPs, this scaling predicts dramatically reduced scattering intensity. Specifically, 14 nm particles are expected to scatter approximately 5 × 10^2^ less light than 40 nm particles and ∼10^4^ less than 65 nm particles, placing their signals below the flow cytometer’s SSC detection threshold. This theoretical consideration supports our experimental observation that 14 nm AuNPs fall below detectable SSC levels (Figure S8). To further validate these results, CLSM confirmed light-scattering signals for 40 and 65 nm AuNPs, whereas signals from 14 nm AuNPs were not detectable under the imaging conditions used (Figure 4A).

We then evaluated silver as an alternative nanomaterial. We selected silver nanoparticles (AgNPs) with an average size of 30 nm. We comprehensively characterized the physicochemical properties of these AgNPs, including size and size distributions before and after PEGylation with 10-kDa PEG (Figure S9). As shown in Figure 4C and 4D, both CLSM and flow cytometry detected these nanoparticles after incubation with RAW 264.7 macrophages. These results suggest that AgNPs exhibit relatively stronger light-scattering properties compared to AuNPs of similar sizes.^64^ To demonstrate that the detection of nanoparticle–cell interactions is not limited to RAW 264.7 macrophages, we tested 4T1 murine breast cancer cells. The results in Figure 4E and 4F highlight that the label-free flow cytometry-based quantification of nanoparticle–cell interactions can be broadly applied to other cell lines and cell types.

We further investigated the effect of surface chemistry on AuNPs interactions with cells by comparing PEGylated and HEP-coated 100 nm AuNPs. We observed that RAW 264.7 cells interacted with HEP-coated AuNPs ∼ 21-fold more compared to PEGylated AuNPs (Figure S10). This result is consistent with our previous studies, which have shown that HEP-coated nanoparticles are efficiently internalized by innate immune cells. Heparosan (HEP) is a polysaccharide ligand that mediates multivalent interactions with cell-surface receptors. Previous studies have shown that the uptake of HEP-coated nanoparticles depends on the surface density of HEP, suggesting that increased receptor engagement enhances cellular internalization. This process has been reported to occur through energy-dependent endocytic pathways, primarily clathrin-mediated endocytosis and macropinocytosis.^15,30,54,65^ These mechanisms provide a plausible explanation for the substantially higher cell internalization of HEP-coated AuNPs compared with PEGylated AuNPs observed in our experiments. To further examine the role of PEG coating on nanoparticle–cell interactions, we compared SSC signals in RAW 264.7 macrophages exposed to PEG-free, 2-kDa PEG-coated, and 10-kDa PEG-coated AuNPs, further confirming that cellular uptake is dependent on nanoparticle surface modification (Figure S11).

### Label-Free Quantification of Nanoparticle-Cell Interaction Kinetics

Next, we applied the label-free flow cytometry workflow to study nanoparticle–cell interaction kinetics. RAW 264.7 cells were incubated with 100 nm AuNPs at four different molar concentrations and analyzed for up to 24 h. As shown in the CLSM images (Figure 5A), the light scattering signals increased with time and concentration, suggesting nanoparticle interactions with the cells. Flow cytometry was then used to monitor these interactions in more detail (Figure 5B, 5C).

**Figure 5:**
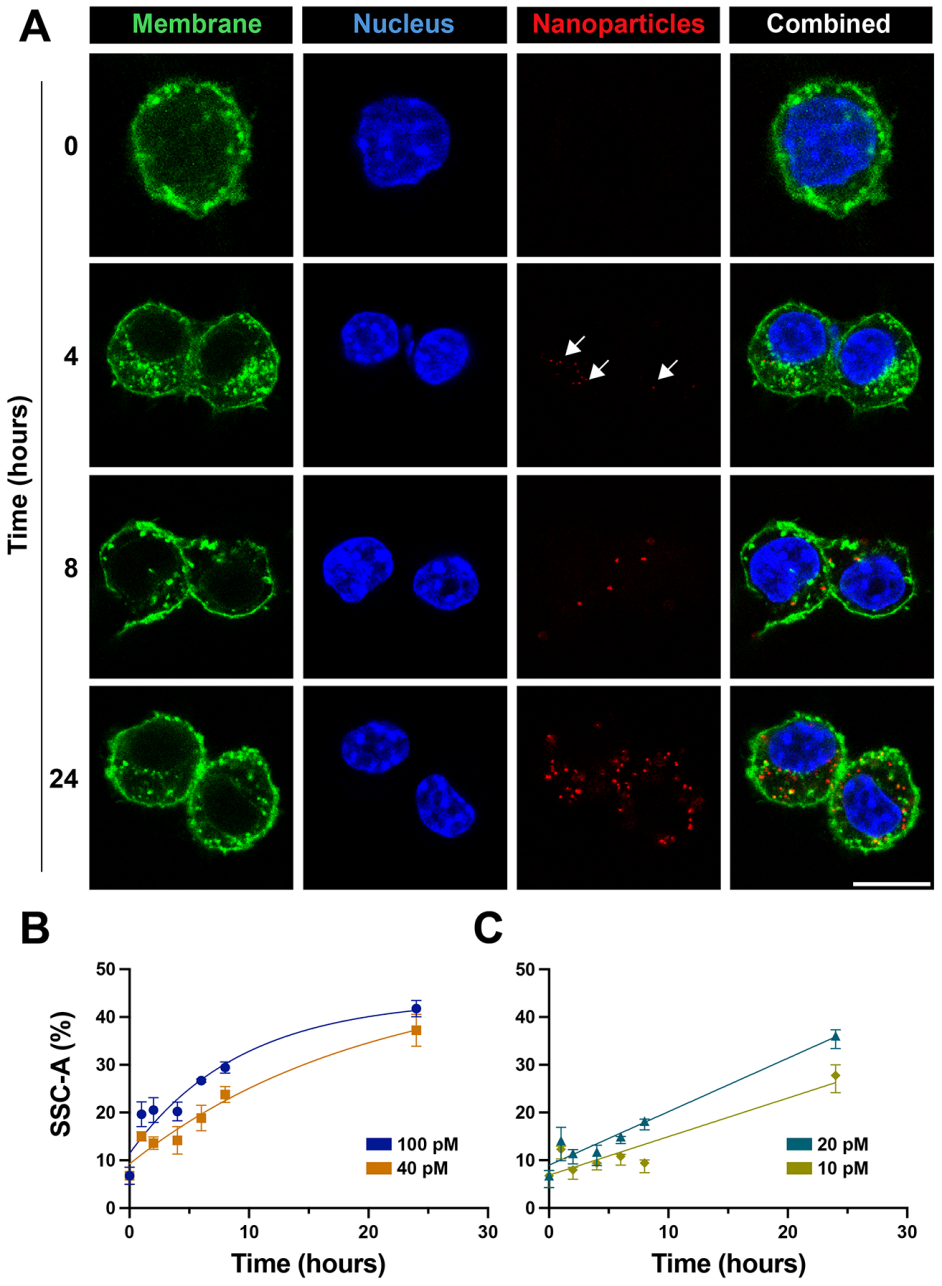
Quantification of nanoparticle–cell interaction kinetics. (A) CLSM images depict the time-dependent interactions of 100 nm gold nanoparticles (AuNPs) with RAW 264.7 murine macrophage cells. The cell nuclei were stained with DAPI (blue), the cell membranes with WGA-CF488A (green), and the red signals indicate nanoparticle light scattering. The scale bar represents 10 μm. The white arrowheads indicate the locations of the nanoparticle signal. (B, C) Flow cytometry quantification of time-dependent nanoparticle–cell interactions at four AuNP concentrations. SSC-A(%) values increased over time in a concentration-dependent manner, reflecting progressive AuNP uptake by RAW 264.7. (B) The higher AuNP concentrations (100 pM and 40 pM) exhibited one-phase association kinetics (solid lines; R^2^ = 0.89 and 0.91, respectively), indicating rapid uptake and clear plateaus characteristic of saturable behavior. (C) The lower AuNPs concentrations (20 pM and 10 pM) exhibited linear trends (R^2^ = 0.92 and 0.83, respectively), indicating slower, unsaturated uptake within the experimental time frame. Data represent mean ± SD (*n* = 3).

At higher nanoparticle concentrations (100 pM and 40 pM), the SSC-A(%) signal followed a one-phase association model, indicating saturable uptake. The model produced strong fits (R^2^ = 0.89–0.91) with clear plateaus, suggesting that uptake progressed rapidly before reaching saturation. The time constants (τ) were 9.3 h for 100 pM and 19.9 h for 40 pM, confirming faster nanoparticle–cell interaction kinetics at higher nanoparticle concentrations.

At lower concentrations (20 pM and 10 pM), the SSC-A(%) signal showed a linear trend (R^2^ = 0.92 and 0.83, respectively), consistent with slower, unsaturated uptake over the experimental time frame. The slopes of the linear fits (1.12 ± 0.07 and 0.81 ± 0.08 SSC-A(%) h^–1^) represent the rate of change in nanoparticle–cell interactions over time. The weaker signal and lack of a plateau likely reflect subsaturating nanoparticle availability and reduced flow cytometry sensitivity due to subtle side-scatter changes at these concentrations.

Batch ICP-MS quantification further demonstrated a time-dependent increase in nanoparticle uptake by RAW 264.7 macrophages (Figure S12).

### Label-Free Quantification of Nanoparticle–Cell Interactions in Mixed-Cell Populations of RAW 264.7 and DC2.4 Cells

To test whether we could use flow cytometry to simultaneously differentiate nanoparticle–cell interactions across various cell types, we incubated RAW 264.7 murine macrophages and DC2.4 murine dendritic cells separately with 100 nm AuNPs. The two cell populations were distinguished using the membrane dyes DiI and DiD, respectively. We selected these cells for their distinct immunological functions and relevance to preclinical research. We then prepared mixtures of RAW 264.7 and DC2.4 cells at defined ratios and analyzed them by flow cytometry (Figure 6). Our gating strategy is depicted in Figure S13 along with the references and control samples in Figure S14.

**Figure 6:**
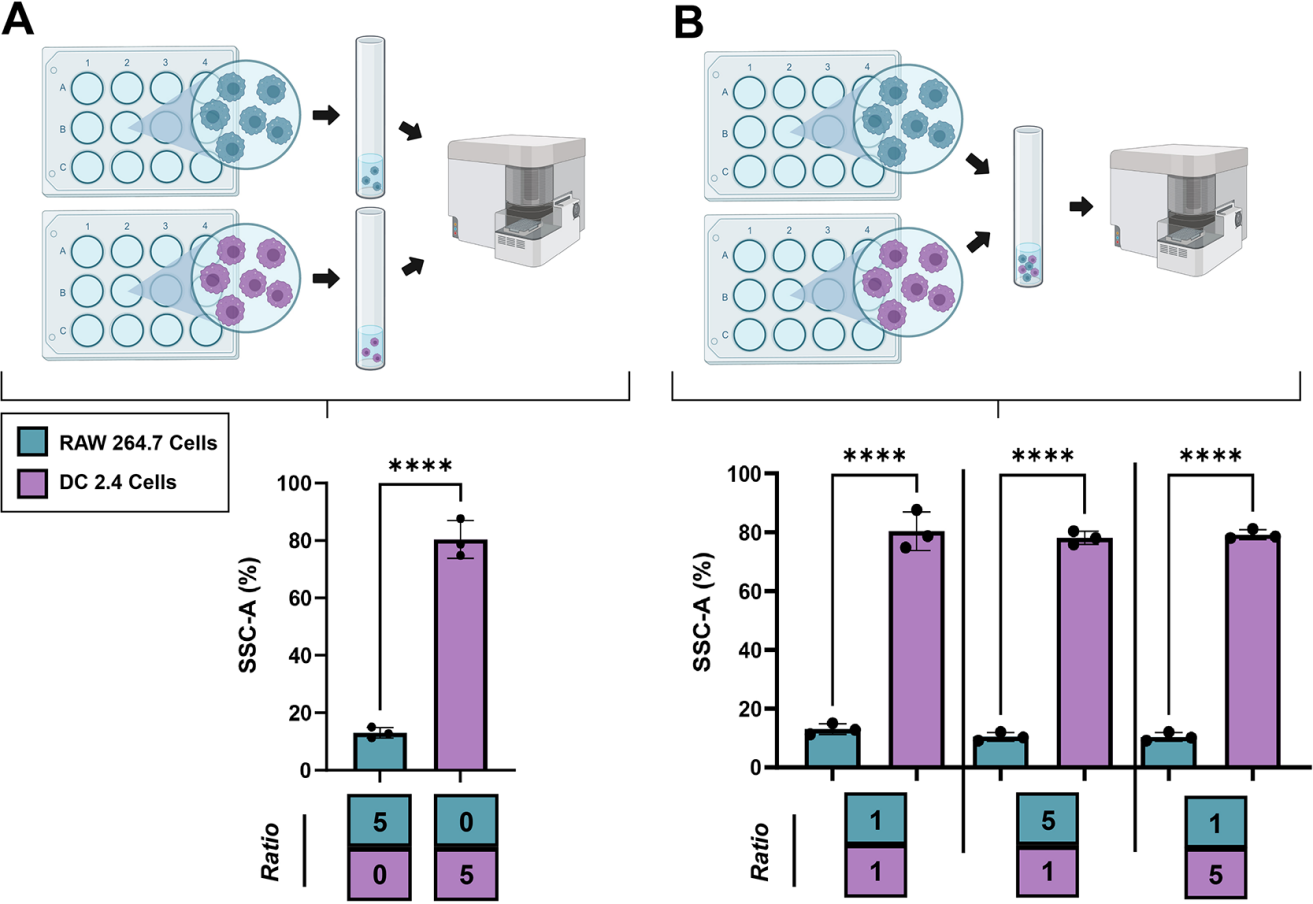
Label-free detection and quantification of gold nanoparticle (AuNP) interactions in mixed-cell populations of murine RAW 264.7 macrophages and DC2.4 dendritic cells. (A) RAW 264.7 and DC2.4 cells were independently treated with 100 nm AuNP (0.1 nM) and analyzed separately by flow cytometry. A significantly higher percentage of DC2.4 cells showed interactions with AuNPs as determined by SSC-A (%) compared to RAW 264.7 cells (****p < 0.0001, unpaired *t* test, *n* = 3, mean ± SD). (B) The RAW 264.7 and DC 2.4 cells were mixed at different cell number ratios of (1:1, 5:1, 1:5). The SSC-A (%) values for RAW 264.7 and DC2.4 remained consistent across all conditions; (****p < 0.0001, one-way ANOVA, *n* = 3, mean ± SD). Created with BioRender.com.

Using this strategy, we observed that DC2.4 cells consistently exhibited significantly higher SSC-A(%) values than RAW 264.7 cells. This finding suggests that a larger cell percentage of the DC2.4 cell population interacts with nanoparticles. To corroborate this finding, we used CLSM imaging and observed that the nanoparticles were more evenly distributed within DC2.4 cells, whereas in RAW 264.7 cells, nanoparticle interactions were more heterogeneous, reflecting varying cell morphologies. For example, more elongated RAW 264.7 cells showed higher nanoparticle interactions than their spherical counterparts (Figure S15).

The ability to detect these differences in nanoparticle–cell interactions at the single-cell level for different cell types is particularly important for targeted nanomedicine applications.^50,53,66^ For example, some cancer nanomedicine treatment strategies require the targeted delivery of nanomedicines within heterogeneous tumor tissues, where macrophages and dendritic cells coexist and actively modulate therapeutic outcomes. These findings establish our label-free flow cytometry approach as a high-throughput method for understanding nanoparticle–cell interactions in relevant mixed-cell model systems.

### Label-Free Quantification of Nanoparticle–Cell Interactions in Coculture Models of RAW 264.7 and DC2.4 Cells

To better reflect the complexity of the in vivo environment, we established a coculture model of RAW 264.7 macrophages and DC2.4 dendritic cells at a 1:3 ratio. We based this ratio on observations that RAW 264.7 cells proliferate approximately 3 times faster than DC2.4 cells over a 48-h incubation period in the previous mixed-cell experiment. We quantified the nanoparticle–cell interactions in each cell type using flow cytometry (Figure 7A, 7B). The gating strategy is shown in Figure S16.

**Figure 7:**
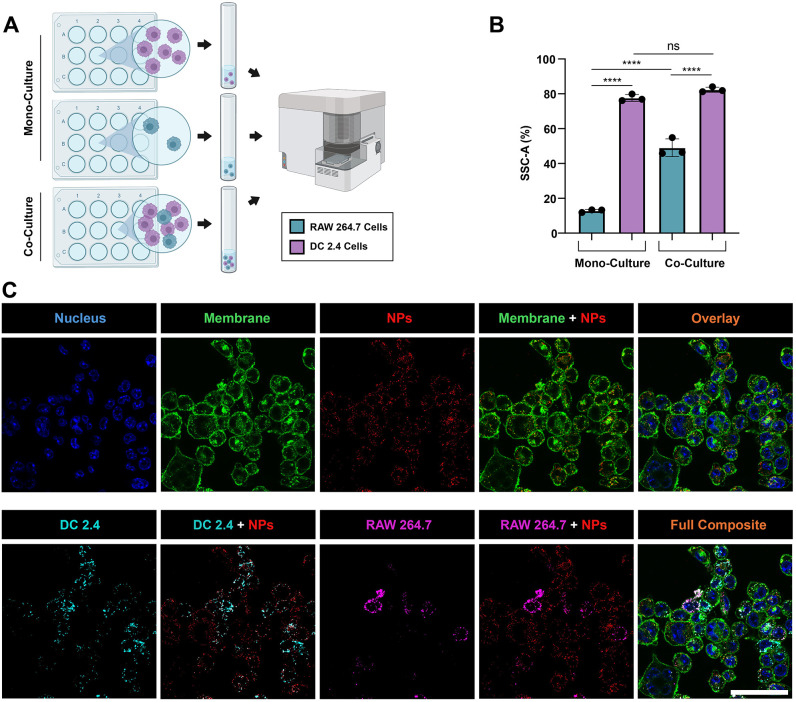
Label-free detection of gold nanoparticle (AuNP) interactions in monoculture and coculture models of murine RAW 264.7 macrophages and DC2.4 dendritic cells. (A) Schematics representing that murine RAW 264.7 macrophages and DC2.4 dendritic cells were cultured either alone (monoculture) or together (coculture), treated with 100 nm AuNPs at 0.1 nM, and analyzed by flow cytometry. (B) The SSC-A(%) values were quantified for each cell population. In coculture, RAW 264.7 cells showed a significant increase in SSC-A(%) compared to monoculture, indicating increased nanoparticle–cell interactions, while DC2.4 cells displayed a nonsignificant change in SSC-A(%); Two-way ANOVA, ns = not significant (*p* > 0.05), ****p < 0.0001, *n* = 3, mean ± SD). (C) Representative CLSM images showing the uptake of AuNPs by RAW 264.7 and DC2.4 cells in the coculture model. The cell nuclei were stained with DAPI (blue), cell membranes with WGA-CF488A (green), the internalized AuNPs were visualized by light scatter signals (red), the RAW 264.7 cells were stained with DiI (magenta), and the DC2.4 cells were stained with DiD (powder blue). The scale bar represents 20 μm. Created in part with BioRender.com.

In this coculture model, we observed that RAW 264.7 cells exhibited a significantly higher SSC-A(%) than in monoculture (*p* < 0.0001), indicating increased nanoparticle–cell interactions, potentially mediated by intercellular interactions with DC2.4 cells. By contrast, the SSC-A(%) of the DC2.4 cells remained largely unaffected (Figure 7B). Studies have shown that DC2.4 cells can secrete various cytokines that can influence the behavior of RAW 264.7 macrophages.^41,67^ For instance, pro-inflammatory cytokines such as IL-6 and TNF-α can be released by dendritic cells, which can activate macrophages and promote their polarization toward an M1 phenotype, characterized by pro-inflammatory responses.

While an overlap between DiD and DiI dyes was visually observed (Figure 7C) using conventional CLSM imaging, our spectral flow cytometry approach precisely detected these events and excluded them using our gating strategy (Figure S16). Some spectral leakage between the DiI and DiD channels was observed in the coculture samples, as shown in Figure 7C. This leakage caused a slight overlap between the fluorescence signals of RAW 264.7 (DiI) and DC 2.4 (DiD) cells. To avoid counting these overlapping signals twice, we used a gating strategy (Figure S16d) that selected only single-stained cells and excluded any double-positive or leaky events from the analysis.

It is important to note that the label-free aspect of this workflow refers specifically to the detection of nanoparticle–cell interactions through intrinsic light-scattering signals, rather than the identification of different cell populations. In the mixed-cell and coculture experiments presented here, fluorescent membrane dyes were used to distinguish RAW 264.7 macrophages from DC2.4 dendritic cells, while nanoparticle detection itself remained label-free through SSC-based measurements. In more complex biological systems, several strategies could be used for cell-type identification while preserving label-free nanoparticle detection. For example, minimal immunophenotyping using a small set of cell-surface markers could be applied to distinguish major cell populations.^68^ Alternatively, genetically encoded reporters or lineage-specific fluorescent proteins could enable cell identification without additional staining during nanoparticle exposure. In some cases, intrinsic scatter properties or morphological differences may also allow partial discrimination of cell populations.^69,70^ However, in highly heterogeneous samples in which scatter properties overlap across cell types, additional labeling strategies may still be required for reliable cell-type assignment.

In summary, building on prior SSC-based studies that demonstrated label-free detection of nanoparticle uptake in single-cell type systems, this work extends the approach into a broader multiparameter workflow that enables more comprehensive characterization of nanobio interactions at the single-cell level.^20,22–27^ This high-throughput approach may be widely used to study nanoparticle–cell interactions in a label-free manner for nanomaterials with relatively high light-scattering properties, such as inorganic nanomaterials made from metals and other composite materials.

## Conclusions

In this study, we established that conventional flow cytometry can be leveraged as a label-free, high-throughput strategy to quantify nanoparticle–cell interactions with single-cell resolution. We demonstrated that side-scatter signals sensitively reflect differences in nanoparticle size, composition, surface chemistry, concentration, and uptake kinetics, and we validated these measurements using high-resolution optical imaging and elemental mass spectrometry. Unlike existing methods that rely on fluorescent labels, destructive preparation steps, or low-throughput microscopy, our workflow preserves nanoparticle surface chemistry, enables rapid analysis of large cell populations, and extends label-free detection to mixed-cell and coculture models. Building on prior SSC-based studies that primarily focused on qualitative detection of nanoparticle presence, genotoxicity screening, or quantitative SSC-ICP-MS calibration for specific nanoparticle–cell line combinations, this work extends the approach into a broader multiparameter workflow that links specific nanoparticle physicochemical parameters to internalization kinetics and cellular behavior in mixed-cell and coculture environments.^20,22–27^

While the approach is most effective for nanoparticles with strong light-scattering properties and may have reduced sensitivity for small nanoparticles, future work will focus on expanding detection to additional materials, integrating multiparameter cytometry, and applying this framework to primary cells and in vivo-derived tissues.

By enabling systematic single-cell screening of nanobio interactions across diverse nanoparticle designs and biological contexts, our workflow offers a practical and scalable approach to the rational engineering of nanoparticles for improved nanomedicine delivery, safety, and translational performance.

## Supplementary Material



## Notes

Additional experimental details, materials, and methods, including nanoparticle synthesis and surface modification protocols, physicochemical nanoparticle characterization using UV–vis spectrophotometry, DLS, TEM, SP ICP-MS, and zeta potential measurements; cell culture and nanoparticle treatment procedures; staining and fixation protocols; flow cytometry gating strategies; and confocal and expansion microscopy images. (PDF)
