# Supplementary material for: Label-Free and High-Throughput Quantification of Nanoparticle–Cell Interactions at the Single-Cell Level with Flow Cytometry
Source: Anal Chem. 2026 Jun 10;98(24):17718–31. doi: 10.1021/acs.analchem.5c08235 (PMC13295095; doi:10.1021/acs.analchem.5c08235)
Supplement: Supplementary file 1 [file ac5c08235_si_001.pdf]

Supporting Information

Label-Free and High-Throughput Quantification of Nanoparticle-Cell Interactions at the Single-Cell Level with Flow Cytometry

Mobina Mohammadnejad[1], Majood Haddad[1,2], Alex N. Frickenstein[1], Arianna Dambold[1], Vinit Sheth[1], Jezan Alexandre[1], Nathan Means[1], James Bowman[1], Kavita Belligund[1], Hunter Moss[1], Jeesoo Park [1], Yuxin He[1], Stefan Wilhelm[1,3,4,5,6*]

[1] Stephenson School of Biomedical Engineering, University of Oklahoma, Norman, Oklahoma, 73019, USA

[2] Oklahoma Medical Research Foundation, Oklahoma City, OK 73104

[3] Institute for Biomedical Engineering, Science, and Technology (IBEST), University of Oklahoma, Norman, Oklahoma, 73019, USAs

[4] Stephenson Cancer Center, University of Oklahoma, Oklahoma City, Oklahoma, 73104, USA

[5] Harold Hamm Diabetes Center, University of Oklahoma, Oklahoma City, Oklahoma, 73014, USA

[6] Materials Science and Engineering Program, University of Oklahoma, Oklahoma City, Oklahoma, 73019, USA

[*]Corresponding Author:

Stefan Wilhelm, Ph.D.
Email: stefan.wilhelm@ou.edu
ORCID: 0000-0003-2167-6221

Table of Contents

1. Materials and Methods 4

1.1. Materials 4

1.2. Gold Nanoparticle Synthesis and PEGylation 5

1.2.1. Glassware Cleaning 5
1.2.2. Synthesis and Characterization of 14-nm Gold Nanoparticle (AuNP) Seeds 5
1.2.3. Synthesis of 40-, 65-, and 100-nm AuNPs 5
1.2.4. PEGylation of Nanoparticles 6
1.2.5. Heparosan Coating of Nanoparticles 7

1.3. Nanoparticle Characterization 8

1.3.1. Dynamic Light Scattering (DLS) 8
1.3.2. Ultraviolet-Visible (UV-Vis) Spectrophotometry 8
Table S1: Surface plasmon resonance (SPR) peak wavelengths and molar extinction coefficients of gold and silver nanoparticles. 9
1.3.3. Transmission Electron Microscopy (TEM) 10
1.3.4. Single-Particle Inductively Coupled Plasma Mass Spectrometry (SP-ICP-MS) 10
Table S2: Summary of nanoparticle diameter calculations based on single-particle ICP-MS data. 11

1.4. Cell Culture and Sample Preparation 12

1.4.1. Cell Lines and Cell Culture Maintenance 12
1.4.2. Cell Seeding 12
1.4.3. Nanoparticle Treatments 12
1.4.4. Fixation and Quenching 13
1.4.5 Staining 13

1.5. Confocal Laser Scanning Microscopy 15

1.5.1. Coverslip Cleaning and Coating 15
1.5.2. Image Acquisition and Analysis 15
1.5.3. Expansion Microscopy 15

1.6. Flow Cytometry 16

1.7. Statistical Analysis 16

1.8. Figure Creation 16

2. Supporting Information Figures 17

Figure S1: UV–vis spectrophotometry characterization of gold and silver nanoparticles. 17

Table S3: Zeta potential characterization of nanoparticles. 18

Figure S2: High-resolution expansion microscopy images. 19

Figure S3: Live/Dead assay results. 20

Figure S4. Comparison of flow cytometry and CLSM-based quantification of nanoparticle uptake across increasing AuNP concentrations. 21

Figure S5: Physicochemical characterization of 14-nm AuNPs. 22

Figure S6: Physicochemical characterization of 40-nm AuNPs. 23

Figure S7: Physicochemical characterization of 65-nm AuNPs. 24

Figure S8: Size-dependent scaling of AuNP light-scattering intensity based on the Rayleigh limit of Mie theory. F 25

Figure S9: Physicochemical characterization of 30-nm AgNPs. 26

Figure S10: Label-free flow cytometry quantification of nanoparticle-cell interactions for nanoparticles with varying surface chemistry. 27

Figure S11: Effect of PEG chain length on AuNP-cell interactions 28

Figure S12: Batch inductively coupled plasma mass spectrometry (ICP-MS) results. 29

Figure S13: Gating strategy for mixed-cell population models of RAW264.7 and DC2.4 cells. 30

Figure S14: References and control samples for the mixed-cell experiment. 31

Figure S15: CLSM image showing heterogeneous nanoparticle-cell interactions. 32

Figure S16: Gating strategy for the co-cultured cell population model. 33

References *34*

1. Materials and Methods

1.1. Materials

10× PBS solution (Bio Basic, PD8117); 1.5H glass bottom dishes (Fisher Scientific, 50-305-807); 12-well cell culture plate (VWR, 10062-894); 18-mm round coverslips #1 (VWR, 16004-300); bovine serum albumin (BSA) (Sigma-Aldrich, A7906); cell scrapers (Fisher, 08-100-241); DC 2.4 mouse dendritic cells (Sigma-Aldrich, SCC142M); DiD (Thermo Fisher, D7757); DiI (Thermo Fisher, D3911); dimethyl sulfoxide (DMSO) (Sigma-Aldrich, D2650); DMEM, high glucose, pyruvate (Thermo Fisher, 11995065); fetal bovine serum (Thermo Fisher, 16000044); FACS tubes (VWR, 89497-818); Flow cytometer (Northern Lights, Cytek Biosciences); FlowJo™ v10.8.1 (BD Life Sciences); gelatin from bovine skin Type B (Sigma-Aldrich, G9391); Ghost Dye™ Violet 510 (Cytek, 13-0870-T100); gold(III) chloride trihydrate (Sigma-Aldrich, 520918); graphpad prism version 10.2.3 (GraphPad Software); glycine (Sigma-Aldrich, 50046); hydrochloric acid (HCl) (Sigma-Aldrich, 320331); hydrogen peroxide (Sigma-Aldrich, 216763); hydroquinone (Sigma-Aldrich, H9003); nitric acid (HNO_3) (Sigma-Aldrich, 438073); NucBlue™ Fixed Cell ReadyProbes™ Reagent (DAPI) (Thermo Fisher, R37606); paraformaldehyde solution (PFA), 4% in PBS (Thermo Fisher, J19943K2); penicillin streptomycin (Thermo Fisher, 15-140-122); RAW264.7 mouse macrophages (ATCC, TIB-71); RPMI-1640 medium (ATCC, 30-2001); sodium bicarbonate (Sigma-Aldrich, S6014); sodium borohydride (Sigma-Aldrich, 213462); sodium citrate tribasic dihydrate (Sigma-Aldrich, S4641); SpectroFlo® software (Cytek Biosciences); sulfuric acid (VWR, JT9681); trypsin-EDTA (Thermo Fisher, 25200072); T-75 cell culture flask (VWR, 734-23131); T-182 cell culture flask (VWR, 734-2315); TWEEN® 20 (Sigma-Aldrich, P9416); 4T1 mouse breast cancer cells (ATCC, CRL-2539); ICP-MS (PerkinElmer NexION 2000); Triton X-100 (Sigma-Aldrich, cat. no. 93426); N,N′-Methylenebisacrylamide (Bis) (Sigma-Aldrich, M7279); Acrylamide Solution, 40% (Sigma-Aldrich, A4058); Methacrolein, 95% (Sigma-Aldrich, 133035); N,N-Dimethylacrylamide (Sigma-Aldrich, 274135); Urea (Sigma-Aldrich, U5378); Sodium Dodecyl Sulfate (SDS) (Fisher Scientific, AC230421000); N,N,N′,N′-Tetramethylethylenediamine (Sigma-Aldrich, T7024); Potassium persulfate (Sigma-Aldrich, 379824) ; Sodium acrylate (AK Scientific, R624); TEM Grid (Ted Pella, 01813-F); 2-kDa mPEG-SH (Laysan Bio); 5-kDa mPEG-SH (Laysan Bio); 10-kDa mPEG-OPSS (Laysan Bio); 13-kDa HEP (Prepared in-house by DeAngelis lab)

1.2. Gold Nanoparticle Synthesis and PEGylation

1.2.1. Glassware Cleaning

The glassware was cleaned using an aqua regia solution made in the 250-ml Erlenmeyer flask, containing a magnetic stir bar. In sufficient quantity to completely submerge the magnetic stir bar (~75 mL), concentrated hydrochloric acid was first added, followed by nitric acid at a 3:1 volume ratio. After ~5 minutes, the mixture resulted in a bubbling yellow solution, which was then left to rest for an additional 20 minutes. During this time, the bubbling slowed, and the color shifted to orange. After transferring the aqua regia into a separate container, the flask was rinsed five times in the fume hood with deionized water to cover the stir bar and completely neutralize the acid mixture. Following this, the flask was washed an additional 15 times with Milli-Q water outside the fume hood.

1.2.2. Synthesis and Characterization of 14-nm Gold Nanoparticle (AuNP) Seeds

The synthesis of 14-nm gold nanoparticles (AuNPs) was carried out using the Turkevich method with slight modifications. Briefly, a citric acid solution was prepared by dissolving 30 mg of sodium citrate tribasic into 1 mL of Milli-Q water.[1] One mL of this sodium citrate solution was added to 100 mL of Milli-Q water in a cleaned 250-mL Erlenmeyer flask and set to boil on a hotplate with a stir speed of 1 at 300°C. After the solution began to boil, 100 µL of 98.5-mg/mL $HAuCl_4$ was pipetted into the flask. The spin speed was then increased to rapidly spin without eliciting splashing, and the solution was left for 7 minutes. During this process, a color change was observed, with the liquid rapidly shifting from clear to dark purple, then to bright, ruby red. At 7 minutes, the flask was rapidly cooled on ice to terminate the reaction.

The 14-nm AuNPs were then characterized at room temperature by measuring the extinction value to calculate the molar concentration via ultraviolet-visible spectrophotometry (UV-vis) on an Agilent Cary 5000 UV-Vis NIR spectrophotometer. We further used dynamic light scattering (DLS) via a Malvern Zetasizer NanoZS to determine the nanoparticles' hydrodynamic diameter and polydispersity index (PDI). The synthesized 14-nm AuNP seeds were then stored at 4°C.

1.2.3. Synthesis of 40-, 65-, and 100-nm AuNPs

The synthesis of AuNPs larger than 14 nm was performed using a protocol adapted from Perrault et al. and guided by predictive synthesis parameters described by Frickenstein *et al*.[2,3] For the synthesis of 40-nm AuNPs, a cleaned Erlenmeyer flask containing 92.405 mL of Milli-Q water was stirred at ~400 rpm. To this, we added 0.942 mL of 25-mM aqueous gold(III)chloride trihydrate, then 0.942 mL of 15-mM aqueous sodium citrate tribasic dihydrate, then 4.769 mL of citrate-coated 2.4-nM 14-nm AuNP seeds, and 0.942mL of 15-mM aqueous hydroquinone. With the addition of hydroquinone, the solution's color changed from light pink to dark red. The reaction was left overnight, and the resulting nanoparticles were characterized the following day using DLS and UV-vis methods as previously described.

Before characterization, to remove excess reactants and smaller AuNPs from new nucleation, 1 mL of 10% (v/v) Tween20 was added for a final ~0.1% concentration, and the AuNPs were centrifuged at 2,500 xg for 180 minutes at 4°C.[2] The supernatant was discarded, and the pellets were resuspended in 0.1% (v/v) Tween 20 and 0.01% (w/v) sodium citrate tribasic dihydrate, then centrifuged again at 2,500 × g for 30 minutes at 4°C. The supernatant was discarded, and the pellets were resuspended in Milli-Q water for a final volume of ~1 mL. The synthesized 40-nm AuNP pellets were stored at 4°C.

Following the same adopted protocol from Perrault *et al.* as described previously, 65-nm and 100-nm AuNPs were synthesized:

For the 65-nm AuNP growth, 0.961 mL of 25-mM aqueous gold(III)chloride trihydrate, then 0.961 mL of 15-mM aqueous sodium citrate tribasic dihydrate, then 1.411 mL of citrate-coated 2.4-nM 14-nm AuNP seeds, and 0.961 mL of 25-mM aqueous hydroquinone were added to 95.705 mL of Milli-Q water in a cleaned flask. The resulting nanoparticles were then centrifuged for the same time and temperature as stated above, but at 1,200 xg.

For the 100-nm AuNP growth, 99.7 L of 25-mM aqueous gold(III)chloride trihydrate, then 0.997 mL of 15-mM aqueous sodium citrate tribasic dihydrate, then 0.305 mL of citrate-stabilized 2.4-nM 14-nm AuNP seeds, and 0.997 mL of 25-mM aqueous hydroquinone were added to 96.7 mL of Milli-Q water in a cleaned flask. The resulting nanoparticles were then centrifuged for the same time and temperature as stated above, but at 7500 xg.

1.2.4. PEGylation of Nanoparticles

PEGylation AuNPs

Based on our previous studies, a density of 7 PEG/nm^2 is desired to stabilize the colloidal dispersion of synthesized AuNPs.[4] To achieve this, the hydrodynamic diameter of the AuNPs synthesized as described previously is measured using DLS. The molar concentration of the AuNPs is determined using the UV-vis spectrophotometer. Once verified, a 1-nM PEG solution was prepared by dissolving measured amounts of 10-kDa mPEG-OPSS (10 mg/mL) or 2-kDa mPEG-SH (2 mg/mL) in Milli-Q water to achieve a final concentration of 1 mM. The AuNPs are then combined with the PEG solution and dispersed by mixing with pipetting up and down and briefly vortexing. The PEGylated-AuNP solution is then left to rest at room temperature for 30 minutes, after which it is centrifuged at 750 xg (for 100-nm AuNPs) at 4°C for 30 minutes. The supernatant is then removed, and the pellet of PEG-coated AuNPs is resuspended in 0.1% (v/v) Tween 20, 0.01% (w/v) sodium citrate tribasic dihydrate solution. After resuspension, the solution is characterized by measuring the molar concentration and hydrodynamic diameter using a UV-vis spectrophotometer and DLS, respectively, and is stored at 4°C.

PEGylation of silver nanoparticles (AgNPs)

A similar protocol to the one above is used for the PEGylation of silver nanoparticles (AgNPs), with the only change being the use of 5-kDa mPEG-SH instead of the PEG solution to coat the surface and stabilize them. The 30-nm AgNPs were also centrifuged at the same temperature and for the same duration, but at 7,500 × g.

1.2.5. Heparosan Coating of Nanoparticles

To test the effect of surface chemistry on nanoparticle-cell interactions, we coated gold nanoparticles (AuNPs) with 13-kDa OPSS-modified heparosan (HEP) polysaccharides following our previously established procedures.[5] Briefly, we selected a density of 7 HEP/nm^2 to stabilize the colloidal dispersion of citrate-coated 100- nm AuNPs. Similarly to PEGylation, we used DLS to determine the hydrodynamic diameter of the AuNPs before and after HEP coating and measured the molar concentration with a UV-vis spectrophotometer. We prepared a 0.01% (w/v) sodium citrate tribasic dihydrate buffer (TCHB) at pH 3. The desired amount of AuNPs to be coated, the calculated HEP-OPSS amount, and the appropriate TCHB buffer were combined with sufficient DI water to yield 1 mL, then left for 5 minutes. Next, we added increments of 3-M NaCl saline solution to achieve a 0.3 M saline concentration. After 20 minutes of incubation, this step was repeated to reach a final saline concentration of 0.7 M. The HEP-coated AuNPs were then purified by centrifugation at 750 ×g for 30 minutes at 4°C and washed three times by removing the supernatant from the pellet, resuspending in DI water, and repeating the centrifugation steps. The HEP-AuNPs were then resuspended in 0.1% (v/v) Tween 20, 0.01% (w/v) sodium citrate tribasic dihydrate solution. After resuspension, the solution was characterized by measuring the molar concentration and hydrodynamic diameter using a UV-vis spectrophotometer and DLS, respectively, and was then stored at 4°C.

1.3. Nanoparticle Characterization

1.3.1. Dynamic Light Scattering (DLS)

DLS analysis was performed to determine the hydrodynamic diameter and polydispersity index (PDI) of nanoparticle samples. Briefly, 3 µL of nanoparticle suspension was diluted into 997 µL of Milli-Q water in a disposable cuvette. The diluted samples were vortexed briefly and allowed to equilibrate at room temperature prior to measurement. DLS measurements were conducted using a Malvern Zetasizer Nano ZS, with each sample analyzed in triplicate. The instrument software provided intensity-weighted size distribution profiles, from which the z-average diameter and PDI were recorded for each sample. Representative distribution plots were generated, displaying intensity (%) as a function of particle diameter (nm).

1.3.2. Ultraviolet-Visible (UV-Vis) Spectrophotometry

(UV-Vis) Spectrophotometry was performed to record the absorbance spectra and estimate nanoparticle concentrations. The same dilution prepared for DLS analysis (3 µL nanoparticles in 997 µL Milli-Q water) was done in a disposable plastic cuvette and analyzed using an Agilent Cary 5000 UV-Vis-NIR spectrophotometer at room temperature. Absorbance spectra were collected from 400 - 700 nm. The molar concentration of nanoparticles was calculated using the Beer–Lambert law (Equation S1) :

$$A = \varepsilon \, c \, l \quad \textbf{(Equation S1)}$$

Where A is the measured absorbance at the surface plasmon resonance (SPR) peak, ε is the molar extinction coefficient for each nanoparticle size, l is the optical path length of the cuvette, and c is the nanoparticle molar concentration. Molar extinction coefficients (ε) used for each nanoparticle size are summarized in Table S1.

Table S1: Surface plasmon resonance (SPR) peak wavelengths and molar extinction coefficients of gold and silver nanoparticles.

Nanoparticle Diameter (nm)	Nanoparticle Type	Surface Plasmon Resonance (SPR) Peak (nm)	Molar Extinction Coefficient (ε) (M^{-1} cm^{-1})
**14**	AuNP	520	$2.95x10^{8}$
**30**	AgNP	409	1.45×10^{10}
**40**	AuNP	529	$8.42x10^{9}$
**65**	AuNP	545	$3.97x10^{10}$
**100**	AuNP	575	$1.57x10^{11}$

Note: The molar extinction coefficients were obtained from Perrault *et al*. for AuNPs and from Paramelle *et al*. (2014) for AgNPs.[2,6]

1.3.3. Transmission Electron Microscopy (TEM)

First, TEM grid preparation and sample loading were performed. We aliquoted 20–100 µL of nanoparticles, diluted to 1,000 µL with 0.1% v/v Tween-20, and centrifuged the diluted nanoparticle solution at an appropriate speed.[3] We removed the supernatant from the centrifuged solutions and placed approximately 5 µL of the concentrated nanoparticle solution onto a copper TEM grid with a copper film (Ted Pella, 01813-F). We used a JEOL-Zeiss 2010F Field Emission TEM with a Direct Electron DE-12 camera to image the nanoparticles. We analyzed the collected images to estimate the nanoparticle diameter distribution and mean nanoparticle diameter using ImageJ.[3,7] Specifically, we estimated nanoparticle diameter by using ImageJ's Analyze Particle feature to approximate the surface area of each nanoparticle. We then calculated the diameter assuming a roughly spherical geometry.

1.3.4. Single-Particle Inductively Coupled Plasma Mass Spectrometry (SP-ICP-MS)

Single-particle inductively coupled plasma mass spectrometry (SP-ICP-MS) measurements were performed using published methods.[3,4,7,8] Briefly, AuNPs were diluted to ~$3x10^{-16}$ M using Milli-Q water. A PerkinElmer NexIon 2000 with a microfluidic sample introduction system was used to measure the mass of individual particles in solution, creating a mass distribution for each measured particle population. Prior to sample measurement, the transport efficiency was measured using Lu^{175}-doped 3-µm polystyrene beads (Fluidigm). Transport efficiency values varied between 50% and 70%, based on environmental conditions. Additionally, a particle calibration curve was measured for each element analyzed (Au or Ag) using synthesized nanoparticle standards whose diameters had been previously quantified by TEM. After mass distribution measurements by SP-ICP-MS, the nanoparticle diameter distribution was approximated by assuming spherical geometry of nanoparticles and using Equation S2:

$$D = \sqrt[3]{\frac{6*m}{\pi*\rho}}, \qquad \textbf{Equation S2}$$

Where m is the nanoparticle mass, ρ is the density of the particle, and D is the diameter of the particle. The results are summarized in Table S2.

Table S2: Summary of nanoparticle diameter calculations based on single-particle ICP-MS data.

**Targeted Nanoparticle Diameter (nm)**	**Element**	**Surface Modification**	**Density ($g\ cm^{-3}$)**	**Observed Nanoparticle Diameter* (nm)**
30	AgNP	Citrate-Coated	10.49	33.7 ± 5.2
40	AuNP	Citrate-Coated	19.32	41.7 ± 5.6 nm
65	AuNP	Citrate-Coated	19.32	67.2 ± 7.2 nm
100	AuNP	Citrate-Coated	19.32	101.1 ± 7.3 nm
100	AuNP	PEG-Coated	19.32	101.3 ± 7.2 nm

*Mean +/- standard deviation

1.4. Cell Culture and Sample Preparation

1.4.1. Cell Lines and Cell Culture Maintenance

RAW 264.7 murine macrophages were maintained in DMEM cell culture media supplemented with 10% fetal bovine serum (FBS) and 1% penicillin/streptomycin. DC 2.4 mouse dendritic cells and 4T1 mouse breast cancer cells were cultured in RPMI-1640 medium supplemented with 10% FBS and 1% penicillin/streptomycin. All cell lines were incubated at 37°C in a humidified 5% CO_2 atmosphere and were routinely passaged upon reaching 80–90% confluency.

For experiments requiring cell harvesting or staining, RAW 264.7 and 4T1 cells were centrifuged at 200 ×g for 5 minutes at 22°C. DC 2.4 cells were centrifuged at 300 ×g for 3 minutes at 22°C.

1.4.2. Cell Seeding

For all experiments, cells were seeded into 12-well plates at a total volume of 500 µL of complete medium per well. RAW 264.7, 4T1, or DC 2.4 cells were seeded alone at either 5×10^4 or 1×10^5 cells per well, as required by each experimental design. For mixed-cell and co-culture experiments, a total of 1×10^6 cells per well were seeded in 1 mL complete media. Plates were incubated at 37°C with 5% CO_2 for 22–24 hours to allow cell attachment.

1.4.3. Nanoparticle Treatments

The molar concentration of each nanoparticle solution was determined by UV-Vis spectrophotometry prior to dilution in complete media. After the initial adhesion period, the media were replaced with fresh complete media containing the indicated type and concentration of nanoparticles. Cells and nanoparticles were then incubated together for 24 hours at 37°C with 5% CO_2 to allow for uptake. For RAW 264.7 uptake experiments, cells were treated with 100-nm PEGylated AuNPs at a final concentration of 0.216 nM in 500 µL per well. In concentration-dependent uptake studies, 100-nm PEGylated AuNPs were used at 0 nM, 0.025 nM, 0.05 nM, 0.075 nM, 0.1 nM, 0.8nM, and 1 nM in 500 µL per well. For size- and type-dependent uptake, RAW 264.7 cells were treated with 40-nm PEGylated AuNPs at 1 nM, 65-nm PEGylated AuNPs at 1 nM, 30-nm PEGylated AgNPs at 0.216 nM, and 100-nm PEGylated AuNPs at 0.216 nM. In cell–type–dependent uptake experiments, 4T1 cells were treated with 100-nm PEGylated AuNPs at a final concentration of 0.216 nM in 500 µL per well. For time-dependent uptake, RAW 264.7 cells were exposed to 100-nm PEGylated AuNPs at 100 pM, 40 pM, 20 pM ,and 10 pM for 0, 4, 8, or 24 hours. In mixed-cell and co-culture models, cells were treated with 100-nm PEGylated AuNPs at 0.1 nM in 1 mL of complete media.

The volume of nanoparticle stock solution required for each experiment was calculated using the standard dilution equation, $C_1V_1 = C_2V_2$, where C_1 and V_1 represent the concentration and volume of the nanoparticle stock solution, and C_2 and V_2 represent the desired final concentration and total nanoparticle volume, respectively. The calculated volumes of nanoparticle stock and complete media were mixed to achieve the desired total volume for each experimental condition.

1.4.4. Fixation and Quenching

Confocal Laser Scanning Microscopy Samples

Following nanoparticle treatment and washing, cells designated for confocal imaging were fixed directly in the well plate with 300 µL of 4% PFA per well at room temperature for 15 minutes. After fixation, wells were washed three times with 1 mL of 1× PBS. To reduce background fluorescence, quenching was performed by adding 1 mL of 1-mg/mL sodium borohydride in 1× PBS per well and incubating at room temperature for 10 minutes. The solution was aspirated and replaced with 1 mL of 100-mM glycine in 1× PBS, followed by a 20-minute incubation at room temperature. The wells were then washed three times with 1 mL of 1× PBS.

Flow Cytometry Samples

After nanoparticle treatment and washing, cells designated for flow cytometry were detached, transferred to FACS tubes, and fixed by adding 300 µL of 4% PFA per tube, followed by incubation on ice for 10 minutes. Following fixation, samples were centrifuged (500 ×g, 5 minutes, 10°C), and the supernatant was discarded. The pellets were then washed twice with 1 mL of 1× PBS (centrifuging at 500 ×g, 5 minutes, 10°C each time) and resuspended for subsequent flow cytometry analysis.

1.4.5 Staining

Ghost Dye™ Violet 510

Cell viability was assessed using Ghost Dye™ Violet 510 in flow cytometric experiments. Ghost Dye was first diluted 1:10 in 1× PBS (10 µL Ghost Dye + 90 µL 1× PBS). After nanoparticle treatment and prior to fixation, cells were resuspended in 300 µL of 1× PBS in FACS tubes. Then, 10 µL of the diluted Ghost Dye solution was added to each tube, mixed thoroughly by pipetting up and down, and incubated on ice for 10 minutes. Following incubation, samples were centrifuged (500 × g, 5 minutes, 10°C) and washed twice with 1× PBS containing 1% BSA before fixation.

DiD and DiI

DiD and DiI dyes were prepared as stock solutions at a concentration of 0.88 mg/mL in DMSO. For cell labeling in co-culture and mixed-cell experiments, RAW 264.7 and DC 2.4 cells were stained with DiI (5 µL per 1×10^6 RAW 264.7 cells in 1 mL of complete media) and DiD (5 µL per 1×10^6 DC 2.4 cells in 1 mL of complete media), respectively. Staining was performed for 20 minutes in the 37°C incubator, followed by three washes with fresh media to remove excess dye.

NHS-ester BP488

For expansion microscopy, pan-staining was performed using NHS-ester dye. An aliquot of 2 mg/mL NHS-ester dye was thawed at room temperature for 30 minutes prior to use. A 1-M sodium bicarbonate solution was freshly prepared by dissolving 840.1 mg sodium bicarbonate in Milli-Q water to a final volume of 10 mL. The staining solution was prepared to a final concentration of 20 µg/mL NHS ester and 100-mM sodium bicarbonate by combining 25 µL of 2-mg/mL NHS ester, 250 µL of 1-M sodium bicarbonate, and 2,225 µL of Milli-Q water. Each expanded gel in a 6-well plate was incubated with 2.5 mL of the staining solution at room temperature, covered in foil, on an orbital shaker for 1.5 hours. After incubation, the staining solution was removed. The gels were washed three times with 1× PBS for 20 minutes each on a shaker, followed by an additional 1-hour wash in 1× PBS or until the gel was visibly clear.

DAPI and WGA

For CLSM imaging, nuclear and membrane staining were performed by incubating samples with DAPI (two drops per 1 mL) and WGA488 (1:200 dilution) in 1× HBSS for 15 minutes on a shaker at speed 2. Following incubation, samples were washed three times with 1× PBS, with each wash lasting 5 minutes on a shaker.

1.5. Confocal Laser Scanning Microscopy

1.5.1. Coverslip Cleaning and Coating

18-mm round glass coverslips were cleaned using freshly prepared Piranha solution. First, two 100 mL and two 250-mL glass beakers were washed with soap and DI water, thoroughly rinsed with Milli-Q water, and dried. To prepare the Piranha solution, 15 mL of 93% sulfuric acid was carefully added to a 100-mL beaker, followed by 5 mL of 30% hydrogen peroxide. The solution was gently swirled while holding the beaker from the top. After 1–2 minutes, the mixture became exothermic, and the beaker felt warm to the touch. Using clean forceps, coverslips were placed into the Piranha solution and incubated for 15 minutes. The solution was then transferred to an empty 100-mL beaker, which was a designated waste container, and the original beaker was filled with Milli-Q water to rinse the coverslips. This rinse step was repeated two more times. Next, the coverslips were sequentially dipped in two fresh 250-mL beakers of Milli-Q water to ensure thorough removal of residual chemicals. Cleaned coverslips were transferred to individual wells of a 12-well plate. The multi-well plate was placed inside a biosafety cabinet and exposed to UV light for 10 minutes with the lid open and inverted. After sterilization, 1 mL of a 2-mg/mL gelatin solution was added to each well, and the plate was incubated at 37°C for 2 hours. The gelatin was then aspirated, and the coverslips were washed three times with 1 mL of 1× PBS to remove any residual gelatin before cell seeding.

1.5.2. Image Acquisition and Analysis

Fluorescence images were taken on a Zeiss LSM 780 CLSM on the Zeiss Zen 2010 software using a 63x oil immersion objective (NA=1.40) for all the experiments and a 40x water immersion objective (NA=1.20) for the Expansion images using a 405-nm diode laser, 488-nm argon laser, and 561-nm diode-pumped solid-state laser. ImageJ was used to adjust the brightness and contrast of each channel and for z-stack 3D imaging.

1.5.3. Expansion Microscopy

Fixed cells were expanded using a Magnify-based protocol.[9] A gelation stock solution containing 0.416 mL N, N-dimethylacrylamide, 3.4 g sodium acrylate, 1.0 g acrylamide, 50 µL of 2% bisacrylamide, 0.1 g sodium chloride, 1 mL 10X PBS, and Milli-Q water to a final volume of 10 mL was prepared, vortexed, and sonicated until dissolved. The solution was purified by centrifugation (1,800 × g, 5 min), and the supernatant was retained. 1 µL methacrolein, 10 µL of 10% TEMED, and 50 µL of 5% potassium persulfate were added per 1 mL of gelation stock, and 90 µL of the mixture was added into a gelation chamber. Gelation was carried out overnight at 37°C in a humidified incubator. Gels were trimmed, separated from coverslips, and incubated in homogenization buffer (2.5 g SDS, 12.012 g urea, 182.65 mg EDTA, 5 mL 10X PBS, water to 25 mL, pH 7.5) for 6 h at 80°C, followed by three 10 min washes with 1X PBS at room temperature and three 10 min washes with 1% PBS-T at 60 °C. Pan staining with NHS-ester BP488 was performed as described above. Gels were expanded in Milli-Q water with three 10-minute exchanges until the expected expansion was achieved.

1.6. Flow Cytometry

Samples were analyzed on a Cytek Northern Lights flow cytometer equipped with three lasers (violet: 405 nm, blue: 488 nm, red: 640 nm). Gain parameters were optimized for each cell type to ensure consistent signal detection across experiments. For RAW 264.7 cells, gain values were set to 30, 40, and 137, and for 4T1 cells to 15, 25, and 112. For both mixed-cell and co-culture experiments, gain settings were 30,30, and 137. These values correspond to FSC, SSC, and SSC-B, respectively. All parameters were held constant within each experimental group to enable direct comparison of scattering profiles. Data were collected using SpectroFlo and analyzed using FlowJo.

1.7. Statistical Analysis

GraphPad Prism 9 was used for all statistical analyses and bar graphs.

1.8. Figure Creation

Figures were created on GraphPad Prism 9 and rendered in Adobe Illustrator.

2. Supporting Information Figures

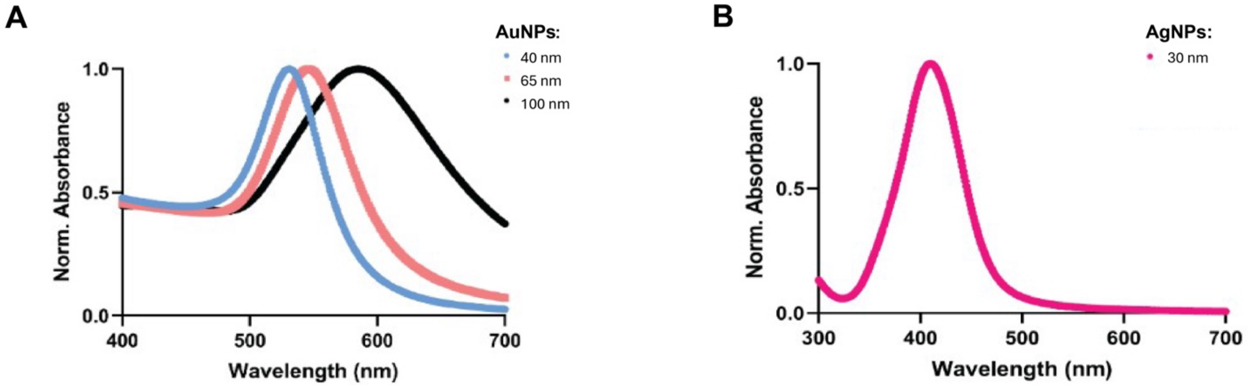

Figure S1: UV–vis spectrophotometry characterization of gold and silver nanoparticles. (A) Normalized absorbance spectra of 40-nm, 65-nm, and 100-nm AuNPs, recorded from 400 nm to 700 nm. (B) Normalized absorbance spectrum of 30-nm AgNPs, recorded from 300 nm to 700 nm. All spectra were normalized to allow visual comparison of absorbance across nanoparticle sizes and materials.

Table S3: Zeta potential characterization of nanoparticles.

Nanoparticle Size (nm)	Material	Surface Modification	Zeta Potential (mV)*
**100**	Gold	Citrate, Tween 20	-26.8 +/- 0.9
**100**	Gold	10-kDa methoxy-PEG-OPSS	1.5 +/- 0.9
**65**	Gold	10-kDa methoxy-PEG-OPSS	1.6 +/- 0.5
**40**	Gold	10-kDa methoxy-PEG-OPSS	-0.2 +/- 0.9
**30**	Silver	5-kDa methoxy-PEG-SH	-4.7 +/- 0.4

*mean +/- standard deviation (n = 3); HEPES buffer (pH 7.4, 10 mM)

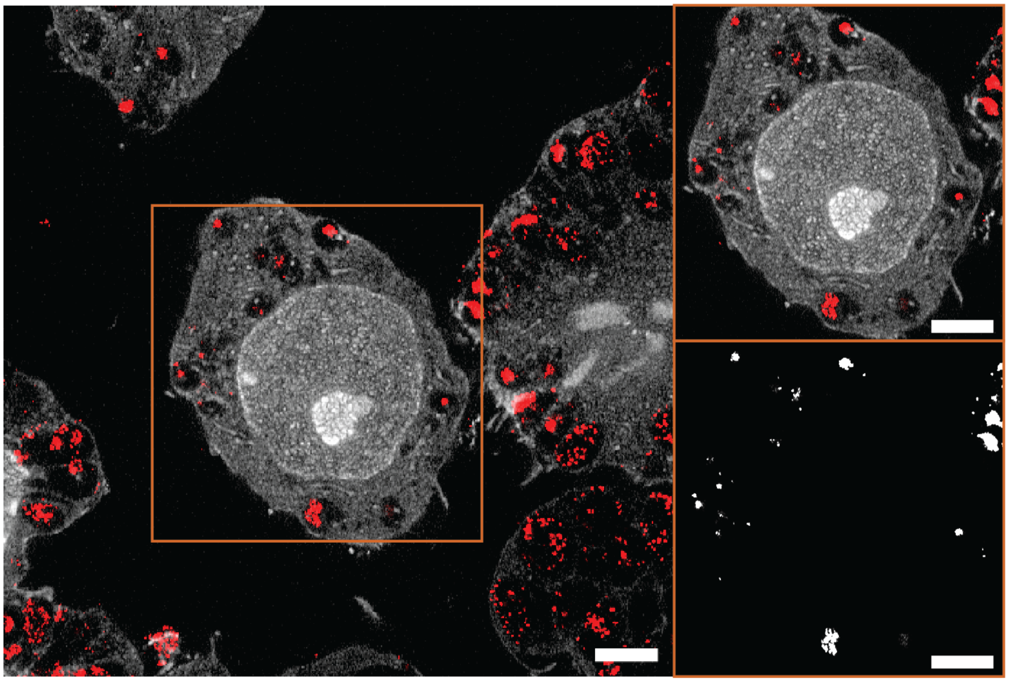

Figure S2: High-resolution expansion microscopy images. RAW 264.7 murine macrophage cells were treated with 100-nm AuNPs at a concentration of 0.1 nM for 24 hours. The images are CLSM micrographs of cells with bulk (pan) staining using fluorescent NHS ester (BP Fluor 488-NHS, grey). Digital magnifications of the regions of interest are shown (orange outline), first with the overlay of both channels (NHS-ester pan stain (grey) and light scattering (red) channels). Next, the nanoparticle light scattering signal is shown in grayscale. The scale bars represent 20 µm.

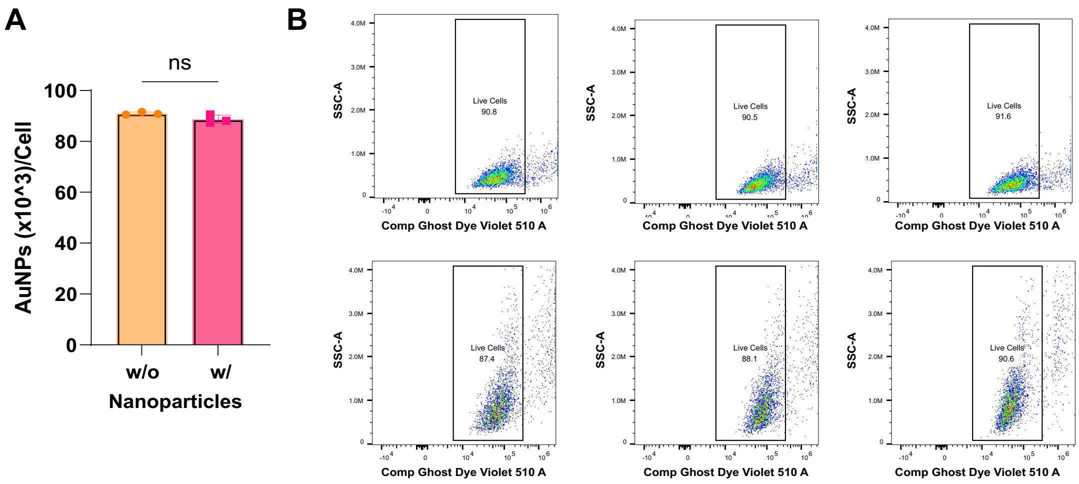

Figure S3: Live/Dead assay results. (A) Quantification of live RAW264.7 macrophage cells shows no significant difference between untreated and AuNPs-treated (0.216 nM, 22 h) samples (mean +/- standard deviation, n=3). (B) Representative flow cytometry dot plots of Ghost Dye Violet 510 staining show comparable proportions of viable (i.e., live) cells in both conditions (top row: untreated; bottom row: AuNP-treated).

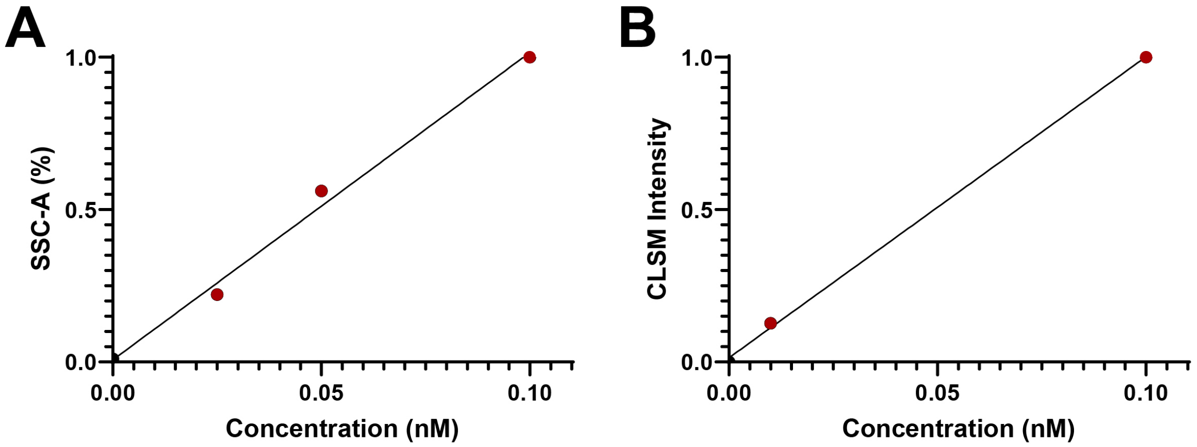

Figure S4. Comparison of flow cytometry and CLSM-based quantification of nanoparticle uptake across increasing AuNP concentrations. (A) Normalized mean SSC-A values measured by flow cytometry in RAW 264.7 macrophages plotted as a function of AuNP concentration (0, 0.025, 0.05, and 0.1 nM), showing a linear relationship ($y = 10.07x + 0.0408$, $R^2 = 0.9926$). (B) Normalized CLSM light scattering intensity quantified from confocal images using ImageJ and averaged across cells at AuNP concentrations of 0, 0.01, and 0.1 nM ($y = 10.61x - 0.0570$, $R^2 = 0.9993$). Despite being derived from independent measurements, both methods exhibit a similar concentration-dependent trend.

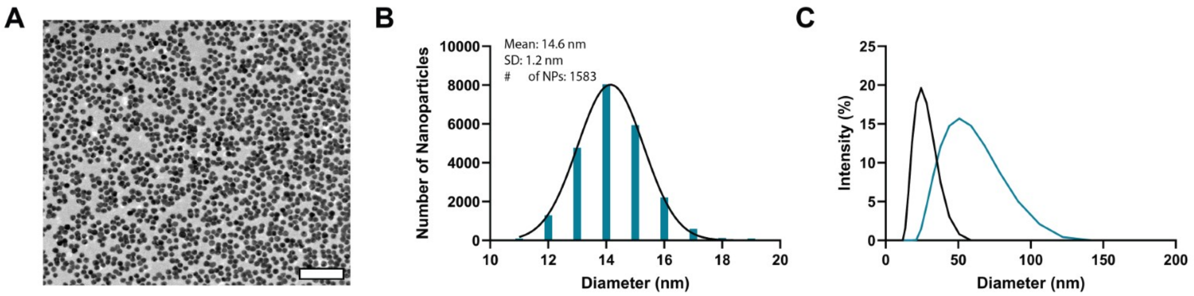

Figure S5: Physicochemical characterization of 14-nm AuNPs. (A) Representative TEM image of synthesized 14-nm AuNPs. The scale bar indicates 100 nm. (B) Nanoparticle size distribution analysis based on TEM images. The size distribution histogram is fitted with a Gaussian curve (black line); mean diameter is 14.6 ± 1.2 nm (n=1583). (C) DLS characterization of citrate-coated 14-nm AuNPs (black, average HDD is 24.2 ± 0.6 nm, PDI = 0.121) compared to 10-kDa PEGylated 14-nm AuNPs (blue, average HDD is 47.5 ± 1.1 nm, PDI = 0.129).

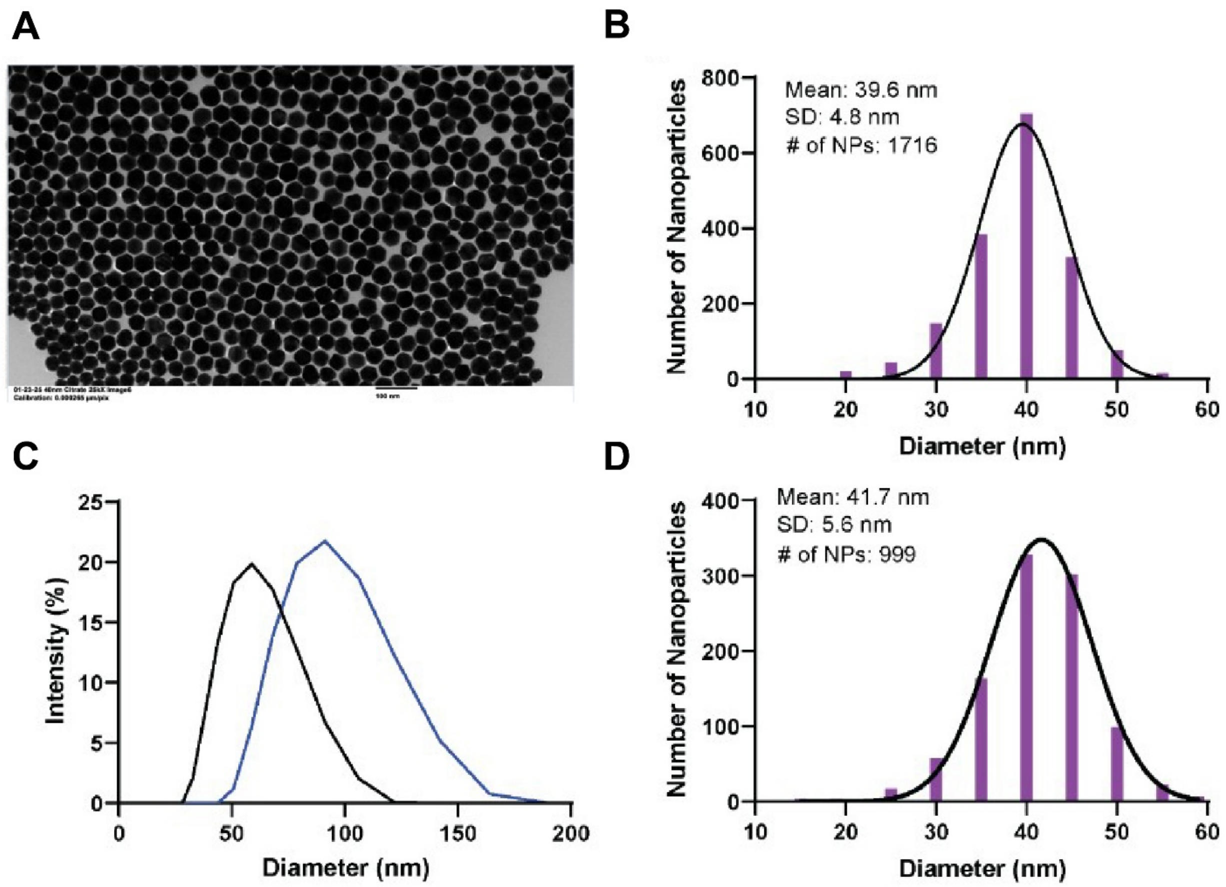

Figure S6: Physicochemical characterization of 40-nm AuNPs. (A) Representative TEM image of synthesized 40-nm AuNPs. The scale bar indicates 100 nm. (B) Nanoparticle size distribution analysis based on TEM images. The size distribution histogram is fitted with a Gaussian curve (black line); mean diameter is 39.6 ± 4.8 nm (n = 1716). (C) DLS characterization of citrate-coated 40-nm AuNPs (black, average HDD is 56.1 ± 1.5 nm, PDI = 0.067) compared to 10-kDa PEGylated 40-nm AuNPs (blue, average HDD is 87.8 ± 1.2 nm, PDI = 0.032). (D) Results of SP-ICP-MS show the core size distributions of citrate-coated (41.7 ± 5.6 nm, n = 999) AuNPs. The black line represents a Gaussian fit.

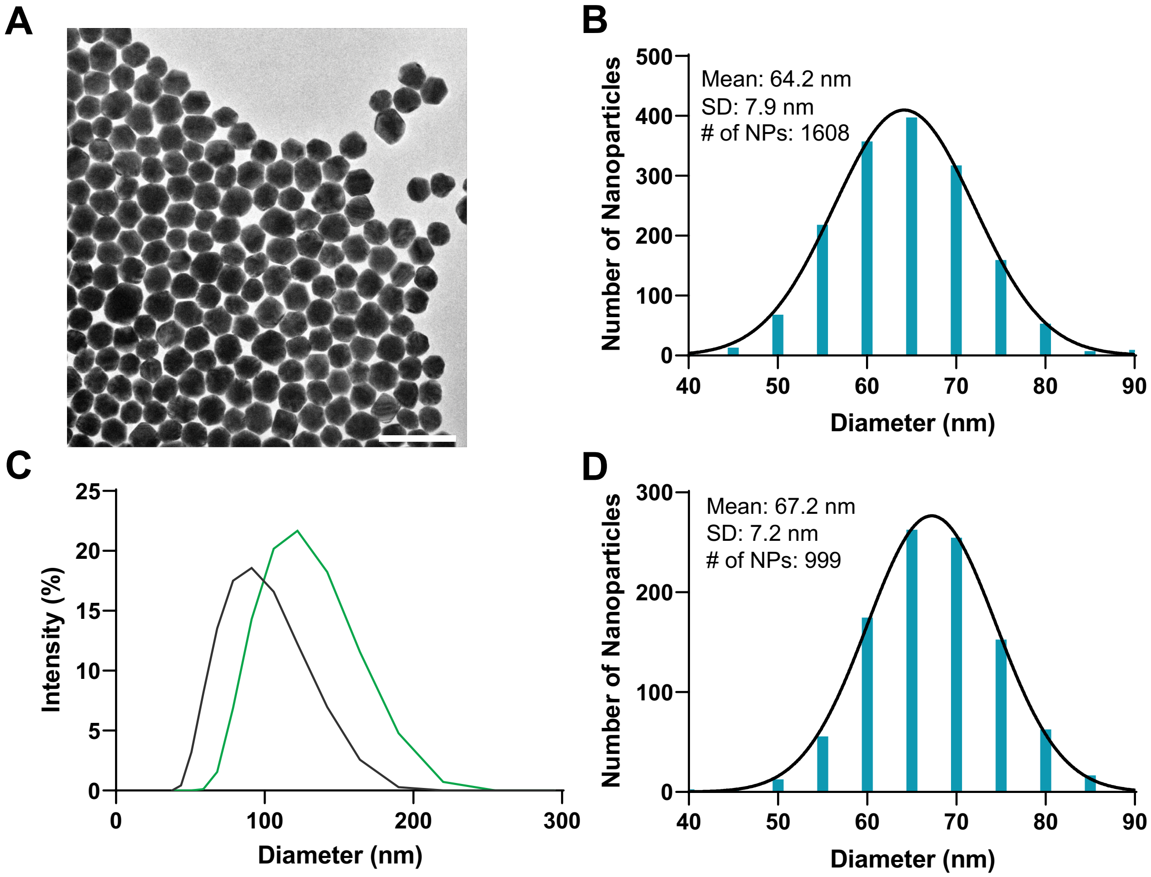

Figure S7: Physicochemical characterization of 65-nm AuNPs. (A) Representative TEM image of synthesized 65-nm AuNPs. The scale bar represents 200 nm. (B) Nanoparticle size distribution analysis based on TEM images. The size distribution histogram is fitted with a Gaussian curve (black line); mean diameter of 64.2 ± 7.9 nm (n=1608). (C) DLS characterization of citrate-coated 65-nm AuNPs (black, average HDD is 86.2 ± 2.2 nm, PDI = 0.065) compared to 10-kDa PEGylated 65-nm AuNPs (green, average HDD is 116.7 ± 2.9 nm, PDI = 0.027). (D) Results of SP-ICP-MS show the core size distributions of citrate-coated (67.2 ± 7.2 nm, n = 999) AuNPs. The black line represents a Gaussian fit.

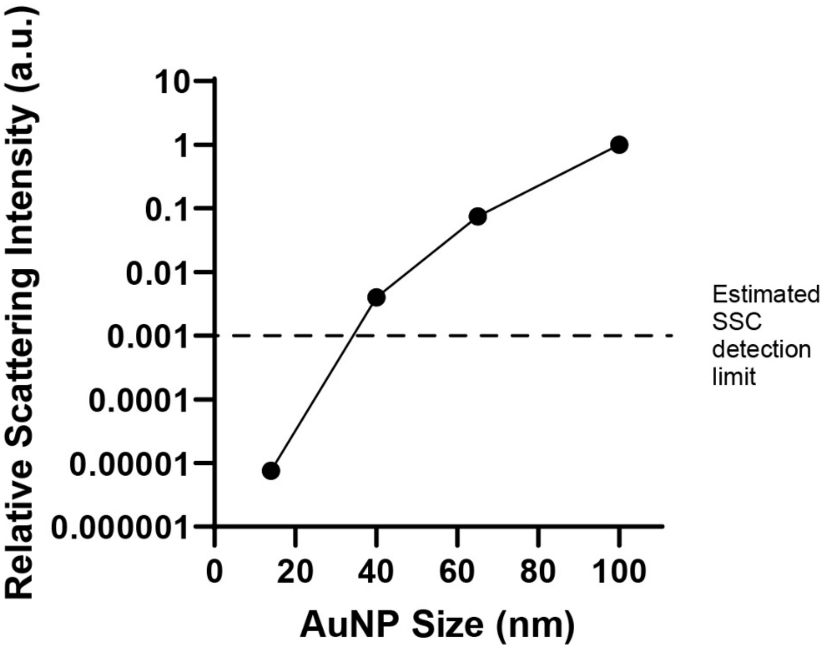

Figure S8: Size-dependent scaling of AuNP light-scattering intensity based on the Rayleigh limit of Mie theory. For particles with diameters substantially smaller than the excitation wavelength, the measured scattering intensity is expected to scale with the sixth power of particle diameter ($I \propto d^6$) under constant illumination. Relative scattering intensities were therefore estimated and normalized to the measured signal for 100-nm AuNPs (set to 1.0). The y-axis is plotted on a logarithmic scale to illustrate the orders-of-magnitude differences in scattering intensity between particle sizes. The dashed horizontal line represents an approximate SSC detection threshold under the experimental conditions used in this study. Because 40-nm AuNPs produced detectable SSC signals, whereas 14-nm AuNPs did not, the detection limit is expected to lie between these particle sizes. To remain conservative, the threshold is placed slightly below the observed scattering intensity of 40-nm particles. This analysis shows a substantial reduction in scattering intensity for 14-nm AuNPs relative to 40-, 65-, and 100-nm particles.

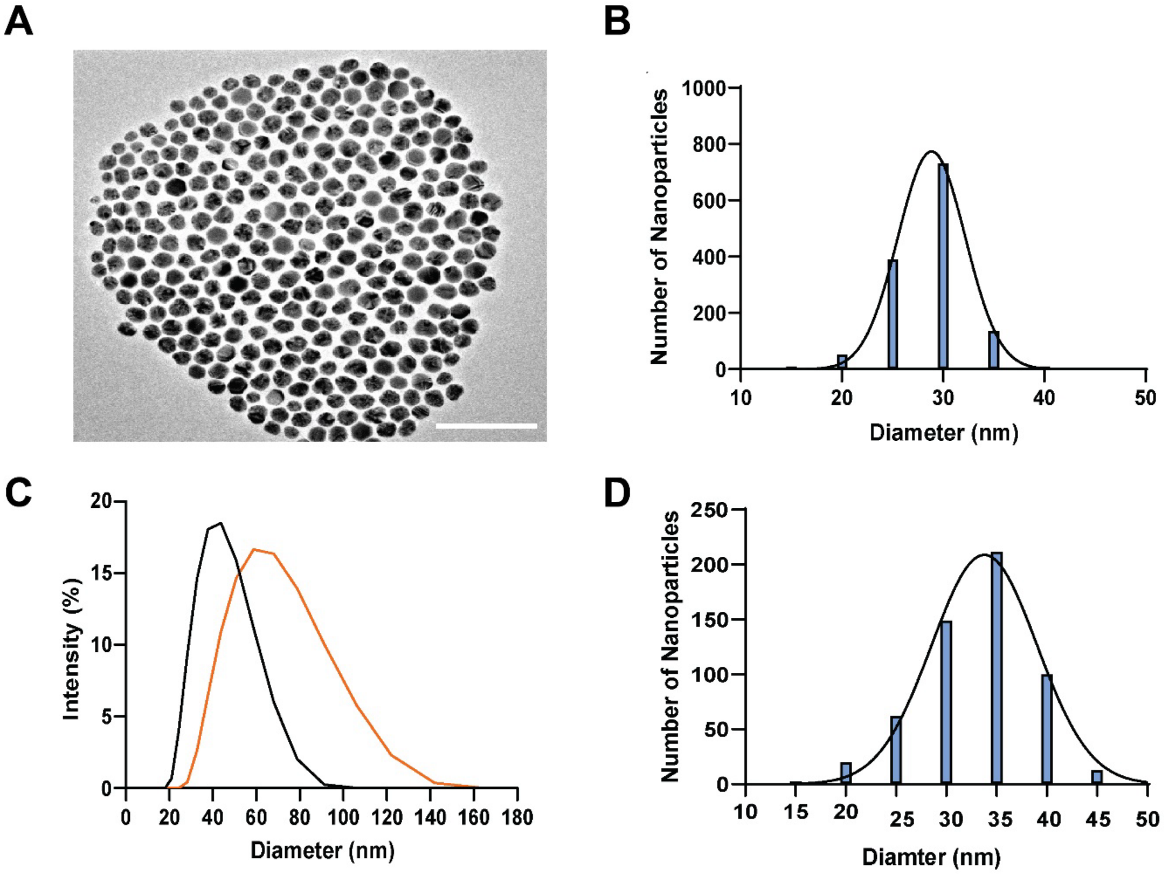

Figure S9: Physicochemical characterization of 30-nm AgNPs. (A) Representative TEM image of 30-nm AgNPs. The scale bar represents 200 nm. (B) Nanoparticle size distribution analysis based on TEM images. The size distribution histogram is fitted with a Gaussian curve (black line); mean diameter is 28.9 ± 3.3 nm (n=1,312). (C)DLS characterization of citrate-coated 30-nm AgNPs (black, average HDD is 38.9 ± 0.1 nm, PDI = 0.116) compared to 5-kDa PEGylated 30-nm AgNPs (Orange, average HDD is 58.6 ± 1.3 nm, PDI = 0.106). (D) Results of SP-ICP-MS show the core size distributions of citrate-coated (33.7 ± 5.2 nm, n=556) AgNPs. The black line represents a Gaussian fit.

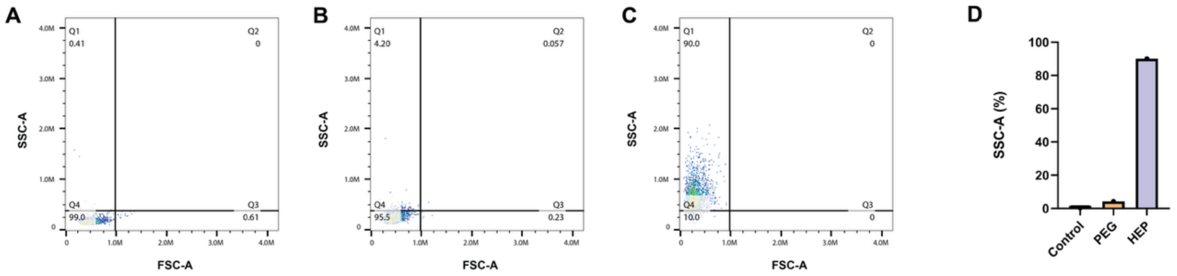

Figure S10: Label-free flow cytometry quantification of nanoparticle-cell interactions for nanoparticles with varying surface chemistry. (A) The dot plots show control RAW264.7 cells (Q1 0.4%), (B) cells incubated with PEGylated gold nanoparticles (Q1 4.2%), and (C) cells incubated with HEP-coated AuNPs (Q1 90%). (D) The SSC-A(%) values confirm that HEP-coated AuNPs exhibited approximately 21-fold higher cell interactions than PEGylated AuNPs.

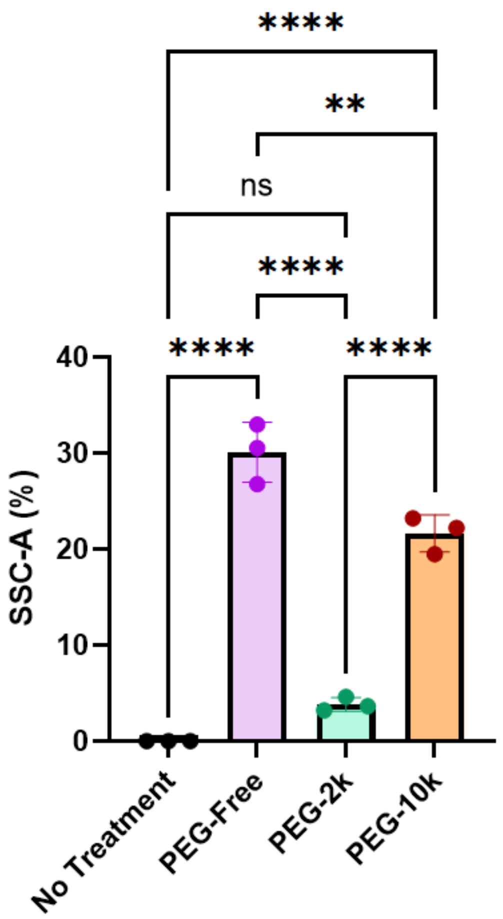

Figure S11: Effect of PEG chain length on AuNP-cell interactions. SSC-A (%) values for RAW 264.7 macrophages exposed to no treatment, PEG-free, 2-kDa PEG-coated (PEG-2k), and 10-kDa PEG-coated (PEG-10k) 100-nm AuNPs (0.216 nM, 24 h). PEG-free AuNPs produced the highest SSC signal, while PEG-2k AuNPs showed no significant difference from untreated cells, indicating strong suppression of cellular interactions by short-chain PEG. PEG-10k AuNPs showed intermediate but significantly elevated uptake compared to both untreated cells and PEG-2k AuNPs. Data represent mean ± SD ($n = 3$). Statistical significance was determined by one-way ANOVA (ns = not significant, ** $p < 0.01$, **** $p < 0.0001$).

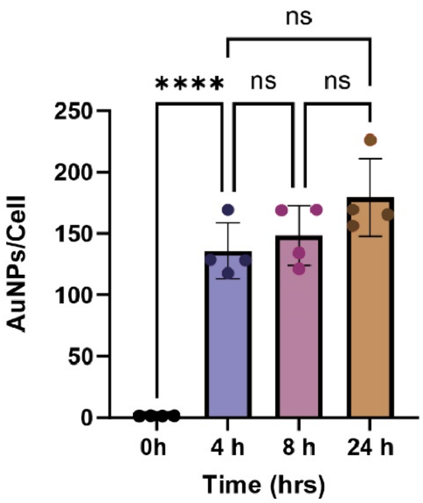

Figure S12: Batch inductively coupled plasma mass spectrometry (ICP-MS) results. The figure shows the uptake kinetics of 100-nm AuNPs by RAW 264.7 cells. One-way ANOVA was performed. ns = not significant, ****$p<0.0001$. The RAW 264.7 cells were treated with AuNPs at a concentration of 0.216 nM (n = 4).

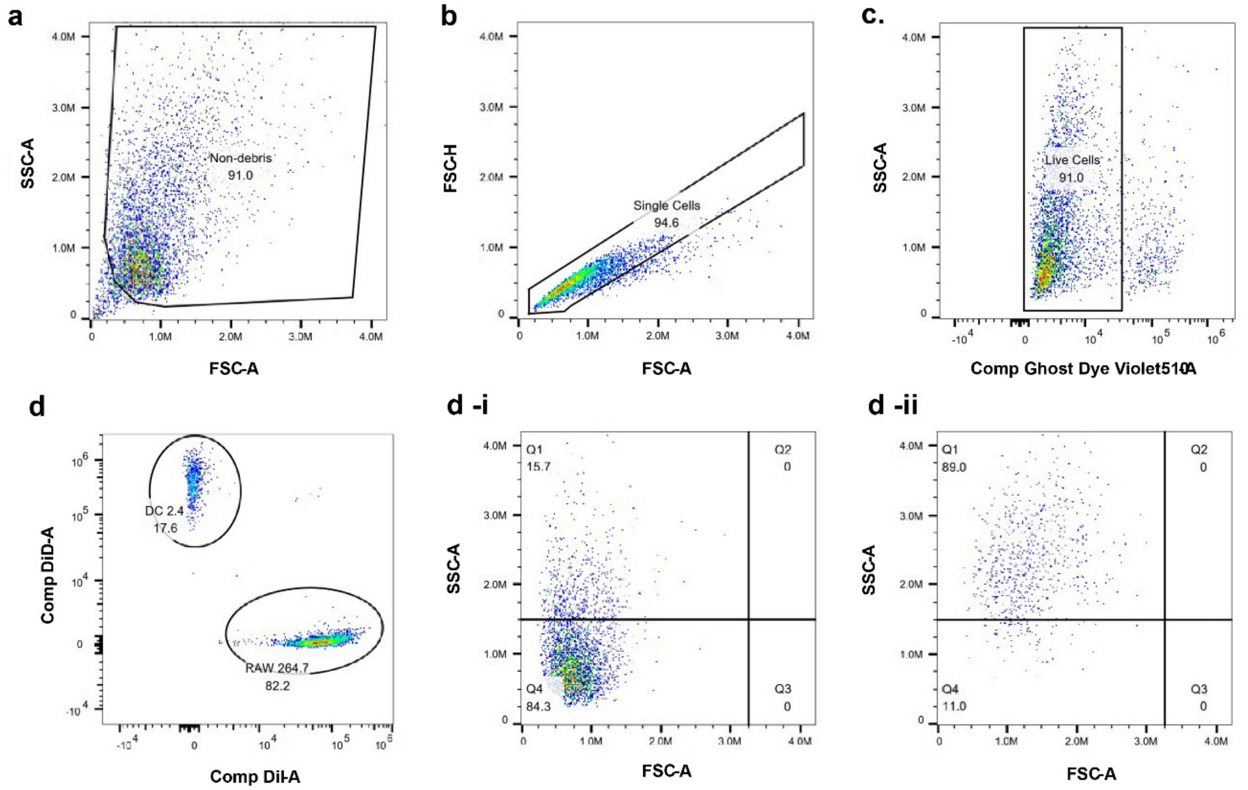

Figure S13: Gating strategy for mixed-cell population models of RAW264.7 and DC2.4 cells. The cells were incubated with 100-nm 10-kDa PEGylated AuNPs. (a) Debris was excluded based on FSC-A vs. SSC-A gating. (b) Singlet events were selected using FSC-H vs. FSC-A. (c) Live cells were gated using Ghost Dye Violet 510-A to exclude dead cells. (d) RAW 264.7 and DC2.4 cell populations were distinguished by lipophilic dye labeling with DiI for RAW 264.7 and DiD for DC2.4. (d-i) The SSC-A(%) values were assessed for RAW264.7 cells from Q1 (d-i), and (d-ii) SSC-A(%) values for DC2.4 cells were assessed from Q1 (d-ii).

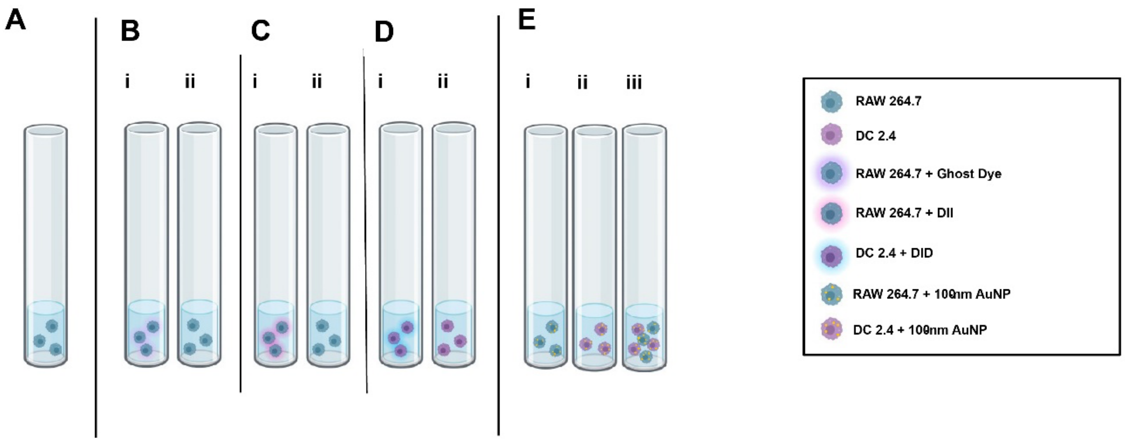

Figure S14: References and control samples for the mixed-cell experiment. (A) Unstained RAW264.7 murine macrophage cells were used as the unstained reference. (B) Ghost Dye Violet 510 viability staining controls: (i) Non-viable cells stained with Ghost Dye Violet 510; (ii) Non-viable, unstained cells used as the negative control for viability dye unmixing under the same conditions. (C) DiI staining controls for RAW264.7 cells: (i) stained with DiI membrane dye (1:200 dilution of 0.88 mg/mL stock in DMSO using complete DMEM; (ii) Unstained universal negative control – unstained RAW264.7 cells processed under the same conditions. (D) DiD staining controls for DC2.4 cells: (i) stained with DiD (1:200 dilution in complete RPMI); (ii) Unstained universal negative control – unstained DC2.4 cells processed under the same conditions. (E) Unstained controls for AuNP treatment groups: (i) RAW264.7 cells treated with 100-nm PEGylated AuNPs without dye; (ii) DC2.4 cells treated with 100-nm PEGylated AuNPs without dye; (iii) mixed population of RAW264.7 and DC2.4 cells treated with 100-nm PEGylated AuNPs (1:1 ratio), without staining. These control and reference samples were used for unmixing in the mixed-cell analysis of nanoparticle-cell interactions by RAW264.7 and DC2.4 cells. The cells were stained with either DiI (RAW264.7) or DiD (DC2.4), and viability was assessed using the fluorescent dye Ghost Dye Violet 510. Created with BioRender.com

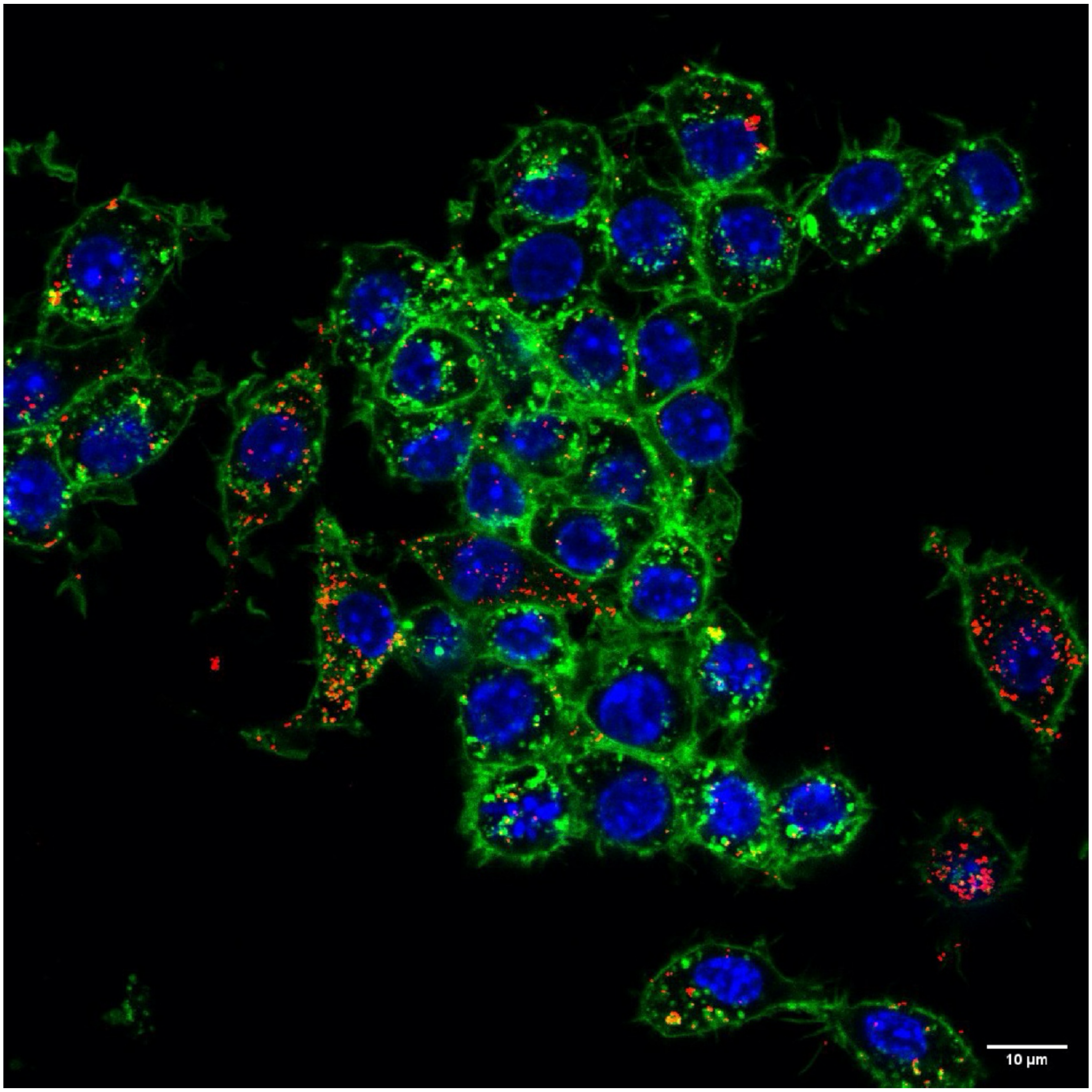

Figure S15: CLSM image showing heterogeneous nanoparticle-cell interactions. RAW264.7 macrophages treated with 100-nm AuNPs at a concentration of 0.1 nM exhibit a heterogeneous distribution of AuNPs. Some cells exhibit substantially increased nanoparticle interactions compared to other cells, as indicated by an increase in the light-scattering signal (red). The cell nuclei were stained with DAPI (blue) and the cell membranes with WGA-CF488A (green). The scale bar represents 10 µm.

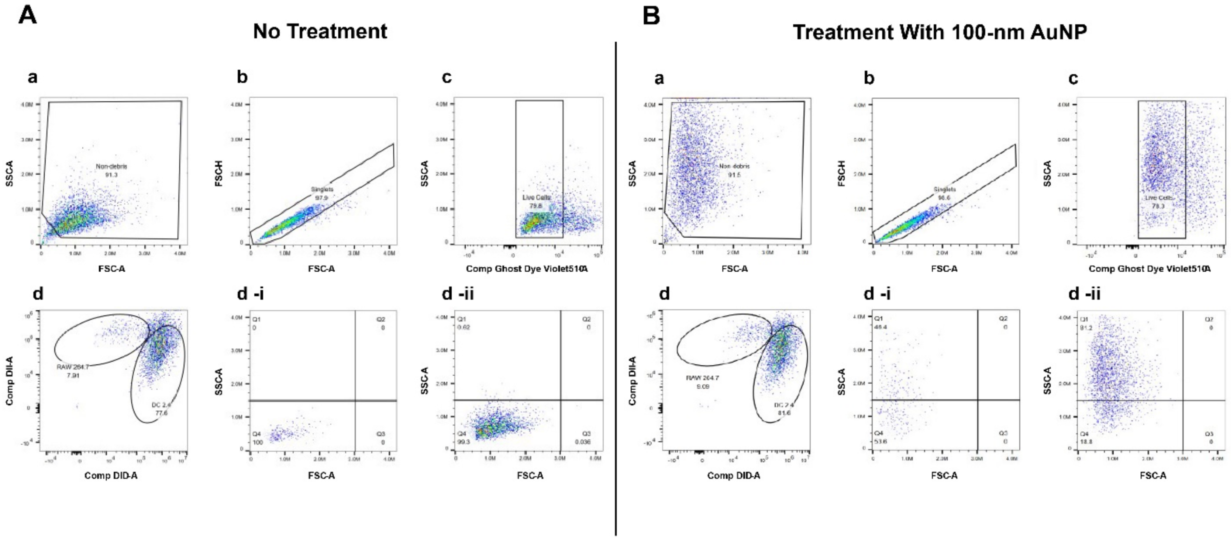

Figure S16: Gating strategy for the co-cultured cell population model. The treatment groups include experiments with and without 100-nm 10-kDa PEGylated AuNPs (0.1 nM). (A) Gating strategy for the untreated co-cultured model of RAW264.7 murine macrophages and DC2.4 murine dendritic cells. (B) Similar co-culture model as (A) but treated with 100-nm 10-kDa PEGylated AuNPs. Note: For both untreated and treated groups (A, B) the following strategy was followed: (a) Debris was excluded based on SSC-A vs FSC-A gating. (b) Singlet events were selected using FSC-H vs. FSC-A. (c) Live cells were gated by excluding dead cells labeled with Ghost Dye Violet 510. (d) RAW264.7 and DC2.4 cell populations were distinguished based on lipophilic dye labeling of DiI for RAW 264.7 and DiD for DC 2.4. (d-i) The SSC-A(%) values were assessed for RAW264.7 cells from Q1, and (d-ii) SSC-A(%) values were assessed for DC2.4 cells from Q1.

References

(1) Turkevich, J.; Stevenson, P. C.; Hillier, J. A Study of the Nucleation and Growth Processes in the Synthesis of Colloidal Gold. *Discuss. Faraday Soc.* **1951**, *11* (0), 55–75. https://doi.org/10.1039/DF9511100055.

(2) Perrault, S. D.; Chan, W. C. W. Synthesis and Surface Modification of Highly Monodispersed, Spherical Gold Nanoparticles of 50-200 Nm. *J Am Chem Soc* **2009**, *131* (47), 17042–17043. https://doi.org/10.1021/ja907069u.

(3) Frickenstein, A. N.; Means, N.; He, Y.; Whitehead, L.; Harcourt, T.; Malik, Z.; Sheth, V.; Longacre, L.; Taffe, H.; Wang, L.; McSpadden, I.; Baroody, C.; Yang, W.; Zhao, Y. D.; Wilhelm, S. The Predictive Synthesis of Monodisperse and Biocompatible Gold Nanoparticles. *ACS Appl. Nano Mater.* **2024**, *7* (19), 23250–23269. https://doi.org/10.1021/acsanm.4c04838.

(4) Frickenstein, A. N.; Mukherjee, S.; Harcourt, T.; He, Y.; Sheth, V.; Wang, L.; Malik, Z.; Wilhelm, S. Quantification of Monodisperse and Biocompatible Gold Nanoparticles by Single-Particle ICP-MS. *Anal Bioanal Chem* **2023**, *415* (18), 4353–4366. https://doi.org/10.1007/s00216-023-04540-x.

(5) Yang, W.; Wang, L.; Fang, M.; Sheth, V.; Zhang, Y.; Holden, A. M.; Donahue, N. D.; Green, D. E.; Frickenstein, A. N.; Mettenbrink, E. M.; Schwemley, T. A.; Francek, E. R.; Haddad, M.; Hossen, M. N.; Mukherjee, S.; Wu, S.; DeAngelis, P. L.; Wilhelm, S. Nanoparticle Surface Engineering with Heparosan Polysaccharide Reduces Serum Protein Adsorption and Enhances Cellular Uptake. *Nano Lett.* **2022**, *22* (5), 2103–2111. https://doi.org/10.1021/acs.nanolett.2c00349.

(6) Paramelle, D.; Sadovoy, A.; Gorelik, S.; Free, P.; Hobley, J.; Fernig, D. G. A Rapid Method to Estimate the Concentration of Citrate Capped Silver Nanoparticles from UV-Visible Light Spectra. *Analyst* **2014**, *139* (19), 4855–4861. https://doi.org/10.1039/C4AN00978A.

(7) Donahue, N. D.; Kanapilly, S.; Stephan, C.; Marlin, M. C.; Francek, E. R.; Haddad, M.; Guthridge, J.; Wilhelm, S. Quantifying Chemical Composition and Reaction Kinetics of Individual Colloidally Dispersed Nanoparticles. *Nano Lett.* **2022**, *22* (1), 294–301. https://doi.org/10.1021/acs.nanolett.1c03752.

(8) Donahue, N. D.; Francek, E. R.; Kiyotake, E.; Thomas, E. E.; Yang, W.; Wang, L.; Detamore, M. S.; Wilhelm, S. Assessing Nanoparticle Colloidal Stability with Single-Particle Inductively Coupled Plasma Mass Spectrometry (SP-ICP-MS). *Anal Bioanal Chem* **2020**, *412* (22), 5205–5216. https://doi.org/10.1007/s00216-020-02783-6.

(9) Klimas, A.; Gallagher, B. R.; Wijesekara, P.; Fekir, S.; DiBernardo, E. F.; Cheng, Z.; Stolz, D. B.; Cambi, F.; Watkins, S. C.; Brody, S. L.; Horani, A.; Barth, A. L.; Moore, C. I.; Ren, X.; Zhao, Y. Magnify Is a Universal Molecular Anchoring Strategy for Expansion Microscopy. *Nature Biotechnology* **2023**, *41* (6), 858–869. https://doi.org/10.1038/s41587-022-01546-1.
